# Smart Adhesives with Multilevel Security Features for Real-World Anticounterfeiting Applications

**DOI:** 10.1021/acsomega.5c07717

**Published:** 2025-12-31

**Authors:** Anand P. J, Namratha Ullal, Dhanya Sunil, Kiran R, Nagabhushana Nayak, Ashok Rao

**Affiliations:** † 125853 Manipal Technologies Limited, Manipal, Karnataka 576104, India; ‡ Department of Chemistry, Manipal Institute of Technology, 76793 Manipal Academy of Higher Education, Manipal, Karnataka 576104, India; § Department of Physics, Manipal Institute of Technology, 76793 Manipal Academy of Higher Education, Manipal, Karnataka 576104, India

## Abstract

Counterfeiting in the manufacturing and publishing industries poses serious threats, including brand damage, financial losses, compromised quality, and infringement of intellectual property rights. Although existing anticounterfeiting technologies like RFID tags, QR codes, holograms, and blockchain offer protection, increasingly advanced counterfeiting methods challenge their effectiveness. Adhesives, despite being essential in manufacturing and bookbinding for their bonding and durability properties, remain underexplored as anticounterfeiting tools. This study presents a novel approach by developing glue formulations embedded with multilevel security markers for real-world applications. Hot melt adhesives are integrated with infrared (IR) emissive and up-conversion green phosphors, offering covert yet verifiable security features while maintaining the adhesive’s key functional properties, including melting point, viscosity, and bonding strength. Under a 980 nm laser light, the secure adhesive exhibits greenish-yellow and red emissions, when viewed without and with a 610 nm band-pass filter, in addition to IR emission at 1020 nm under a 340 nm UV light, detectable by IR cameras or readers. The multisecure glue shows bright green and pink emissions upon a 980 nm illumination along with IR signals similar to the secure adhesive. These hidden yet easily verifiable features provide a promising, tamper-resistant solution to counterfeiting, particularly for book authenticity, enabling discrete verification by authorized users while remaining undetectable to counterfeiters.

## Graphical abstract

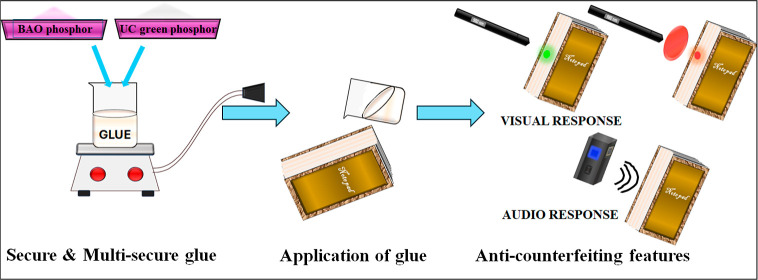

## 1 Introduction

The counterfeiting of goods across industries such as automotive, construction, electronics, books, packaging, graphic arts, pharmaceuticals is a growing problem that triggers serious financial, legal, and reputational issues for companies and consumers alike.^1–4^ The counterfeit products lead to loss in reputation of original brand, proves hazardous to consumer health, and subjects inventors to significant financial loss.^5–7^ Traditional methods such as holograms, watermarks, ultraviolet (UV)–visible inks, tamper-evident seals, taggants and microtexts assist in authenticating genuine products and documents.^8–15^ Digital solutions including QR codes, RFID tags, and blockchain-based tracking offer more advanced, real-time verification and traceability.^16–19^ However, counterfeiters continue to evolve, often replicating visible features or hacking digital systems. Thus, a serious threat to industries, economies, and consumer safety due to counterfeiting demands robust multilayered and intelligent anticounterfeit technologies that combine physical, chemical, and digital tools to stay ahead of sophisticated fraud.

Adhesives or glues represent an underexplored avenue for incorporating anticounterfeit measures and can be readily applied at an industrial scale to safeguard products against forgery. Glues are used in nearly every product in our daily life spanning across a wide range of industries including construction, furniture, packaging, printing, publishing, electronics, electrical, automotive, textile, leather, medical, pharmaceutical, manufacturing, and aerospace, all serving the basic purpose of bonding materials together.^20–22^ Different types of glues including water-based, hot melt, epoxy, pressure-sensitive and UV-curable are chosen based on specific application offering structural strength, flexibility, sealing, or even security.^23^ While conventional adhesives serve basic bonding functions, they lack security features such as tamper indication or authentication capability. Often regarded as a simple and overlooked commodity, glue plays a crucial, yet underappreciated, role in combating counterfeit products. Substituting regular adhesives with security glues can facilitate product safety through (i) difficult to replicate features without specialized knowledge or tools, (ii) enhanced supply chain visibility and product traceability, and (iii) compliance with regulatory and quality standards.^22^ Thus, security glues can serve as a key component of modern anticounterfeiting and tamper-proofing strategies. Their integration across various sectors helps to ensure that only genuine, untampered products reach consumers and regulators.

As counterfeiting becomes more sophisticated, the use of smart adhesives continues to grow in importance. In this context, specialized glues can be incorporated as part of an integrated security system designed to make it more challenging for counterfeiters to reproduce authentic products. Phosphors are widely used in such applications due to their tunable luminescence features. Mechano-luminescent Mn^2+^/Bi^3+^/Er^3+^-doped BaZnOS films and photochromic CaWO_4_/Yb^3+^, Er^3+^, Bi^3+^ inks produce multicolor emissions under specific UV and near-infrared (NIR) excitations.^24,25^ Similarly, cadmium and hybrid metal halides enable hidden, multilevel security features, while fluorescent epoxy adhesives with coumarin and microcrystalline cellulose provide sustainable, covert markings for cultural and antique objects.^26–28^ Previous generations of anticounterfeiting adhesives have primarily relied on simple, single-layer security features, often limited to fluorescent dyes or single-mode luminescence. Among adhesive types such as drying adhesives, hot-melt adhesives, pressure-sensitive adhesives, and reactive adhesives, very few studies have explored hot-melt systems due to the difficulty of incorporating stable anticounterfeiting agents that can withstand their high processing temperatures. In the present work, we demonstrate a next-generation approach by embedding carefully selected phosphors into hot-melt adhesives to achieve multilevel and multimodal luminescent security features without compromising adhesive performance. This strategy not only addresses the material limitations of hot-melt adhesives but also provides a durable, covert, and tamper-resistant anticounterfeiting solution suitable for everyday applications. A commercially available green emissive JUP-AS120 pigment and lanthanide (Ln^3+^) ions-doped barium aluminum oxide (BAO) phosphor are infused in hot-melt glue to develop adhesives with both visual and auditory signals for product authentication. These specialized adhesives can be incorporated into packaging, labels, or product components to authenticate and protect them against forgery. Such intricate security features remain unique, hidden, and hence unknown to the forger, and they fail to ideally replicate them. As a real-world example, the widespread issue of unnoticed counterfeit books, which are often rebranded and sold, causing significant revenue loss to publishers and authors, is addressed in this study. The modified security glue is used for binding a book, and the incorporated security features enabling authentication are demonstrated.

## 2 Materials and Methods

JUP-AS120 was procured from Shenyang Joinunion Chemical Technology Co. Ltd. Swifttherm 8440 procured from Fuller Co. was used as the base adhesive. All raw materials used for the synthesis are of analytical grade.

### 2.1 Synthesis of Lanthanide (Ln^3+^)-Doped BAO Phosphors

Stoichiometric amounts of barium carbonate (BaCO_3_, Molychem India LLP) and aluminum oxide (Al_2_O_3_, SRL chemicals) were ground in mortar and pestle for 10 min. Further, different rare earth oxides in their respective mol %: gadolinium­(III) oxide (Gd_2_O_3_, Sigma-Aldrich, 8.14%), erbium­(III) oxide (Er_2_O_3_, Otto chemie, 7.74%) and ytterbium­(III) oxide (Yb_2_O_3_, Sigma-Aldrich, 6.32%) were added to the above mixture. Further, europium­(III) oxide (Eu_2_O_3_, Sigma-Aldrich) of varying doping concentrations (1.60–15.45 mol %) was added and ground well for 30–45 min. The contents were transferred into different crucibles and placed in a preheated muffle furnace. The mixture was initially heated to a temperature of 800 °C for 4 h. After natural cooling to room temperature, the material was reground to ensure homogeneity and then subjected to a second calcination at 1300 °C for 6 h to complete the formation of the desired phase. Following furnace cooling, the resulting powders were collected and prepared for various characterizations.

### 2.2 Characterization of Ln^3+^ BAO Phosphors

The X-ray diffraction (XRD) patterns of the phosphors were obtained in the range of 15°–80° using a Rigaku MiniFlex benchtop X-ray diffractometer. The diffuse reflectance spectroscopic (DRS) data for the phosphors were recorded in a Jasco V-770 UV–visible/NIR spectrophotometer equipped with a PMT detector at a scan speed of 400 nm/min with UV–vis and NIR spectral bandwidth of 5.0 and 20.0 nm. The emission spanning 400–800 nm was recorded using a Horiba Fluorolog-QM-75–21 spectrofluorometer. The experiments were performed with a 980 nm DPSS CW laser (excitation source) with variable power (0–2 W). The emission profiles in the NIR region were recorded using an InGaAs detector (800–1550 nm) cooled with liquid N_2_ under the same excitation source. The scanning electron microscopic (SEM) images of JUP-AS120 and doped BAO phosphors were obtained using a ZEISS EVO MA18 instrument equipped with an Oxford energy-dispersive spectroscopic (EDS) instrument for elemental composition analysis. The X-ray photoelectron spectroscopic (XPS) data were acquired using Thermo-Scientific K-Alpha system with a X-ray spot size of 400 μm.

### 2.3 Preparation of the Adhesive Formulation

Two types of security adhesives were developed: a secure adhesive containing JUP-AS120 and a multisecure adhesive incorporating both JUP-AS120 and Ln^3+^-doped BAO. To obtain the secure glue formulation, about 9.8 g of Swifttherm 8440 hot melt adhesive granules was placed in a Borosil Glass Petri dish kept on the electrical heating plate and heated to 140 °C. As the granules begin to melt, 0.2 g of JUP-AS120 was added and stirred using a glass rod to obtain the homogeneous secure adhesive formulation. A multisecure adhesive was prepared by incorporating a well ground mixture of JUP-AS120 and Ln^3+^-doped BAO phosphors to the hot melt of the commercial adhesive. About 9.5 g of Swifttherm 8440 hot melt adhesive granules was heated at 140 °C in a Petri dish. JUP-AS120 (0.2 g) and Ln^3+^-doped BAO composite (0.3 g) powders were taken in a mortar and ground well for 10 min to obtain a homogeneous dry mixture. This solid solution was added to the molten hot melt adhesive and stirred with a glass rod to obtain a homogeneous multisecure adhesive formulation.

### 2.4 Viscosity, Peel Resistance, Thermal and Photo Stability Tests of Adhesives

The viscosities of the control, secure, and multisecure adhesives were measured using Brookfield Ametek Viscometer (DVEELVTJ0). The melted glue was poured into the sample container and subjected to different shear rates (10–100 rpm) with the help of 31 Spindle (rotating element) under different temperature conditions. The peel test of the adhesives was achieved using a Peel Adhesion/Bond/Seal Strength Tester (Presto Stantest Pvt. Ltd.). The adhesive laminated paper sample was securely clamped between two jaws of the tester, and a controlled force was applied until it separates or peels off from the adhesive layer. Thermal stability of the adhesives was assessed using a PerkinElmer DSC 6000 instrument at a heating rate of 10 °C/min. The photostability of glue was analyzed using Hari Impex LAB UV 83002 equipment.

### 2.5 Application of Adhesives to the Spine of a Book

Hot molten adhesive was poured over the spine of a book made with maplito paper and evenly spread using a metal spatula. A cover was then placed for binding, and the assembly was allowed to cool at room temperature.

## 3 Results and Discussion

### 3.1 Characterization of JUP-AS120

Initially, the commercially obtained pigment was subjected to SEM–EDS and XPS measurements for elemental analysis. The SEM micro images for commercial JUP-AS120 as presented in Figure 1a,b display particles of a porous nature with well-defined boundaries. The EDS plot as depicted in Figure 1c shows the presence of oxygen (O), ytterbium (Yb), erbium (Er), F (Fluorine), sodium (Na), and yttrium (Y) as elements. The XPS survey (Supporting Information Figure S1a) reveals the presence of F (43.19%), Na (13.7%), and Y (10.71%) in abundance followed by Yb (0.29%). Er content remains undetected, presumably due to the dominance of spectral peaks of Y (150–167 eV) and Yb (180–196 eV) in the survey. The high-resolution spectra are also acquired for Na, Y, and F (Figure S1b–d), while Yb (Figure S1e) and Er (Figure S1f) are present in +3 oxidation state.^29^ The XRD spectrum (Figure S2) presents sharp peaks, indicating the crystallinity of the commercial pigment. The interplanar distance calculated using Bragg’s equation for the JUP-AS120 is shown in Table S1. The crystallite size of the pigment is estimated to be ∼14 μm using a Debye–Scherrer equation $D=\frac{k\lambda }{\beta \cos\;\theta }$ , where the value of *k* is 0.9, λ refers to the source wavelength, β is the full width at half-maximum of the diffraction peak in radians, and θ is Bragg’s angle.

**Figure 1:**
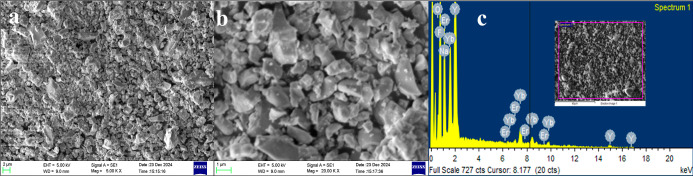
(a) Lower and (b) higher magnification SEM images and (c) EDS spectrum depicting chemical composition of JUP-AS120 (inset shows the area of interest).

JUP-AS120 exhibits IR emission upon excitation at 340 nm (Figure 2a). Further the phosphor displays a dominant green upconversion (UC) emission at 540 nm along with minor emissions at 522, 530, 660, and 670 nm (red) at 980 nm excitation as presented in Figure 2b. In addition, IR emission was also observed under the same excitation conditions, as shown in Figure 2c.

**Figure 2:**
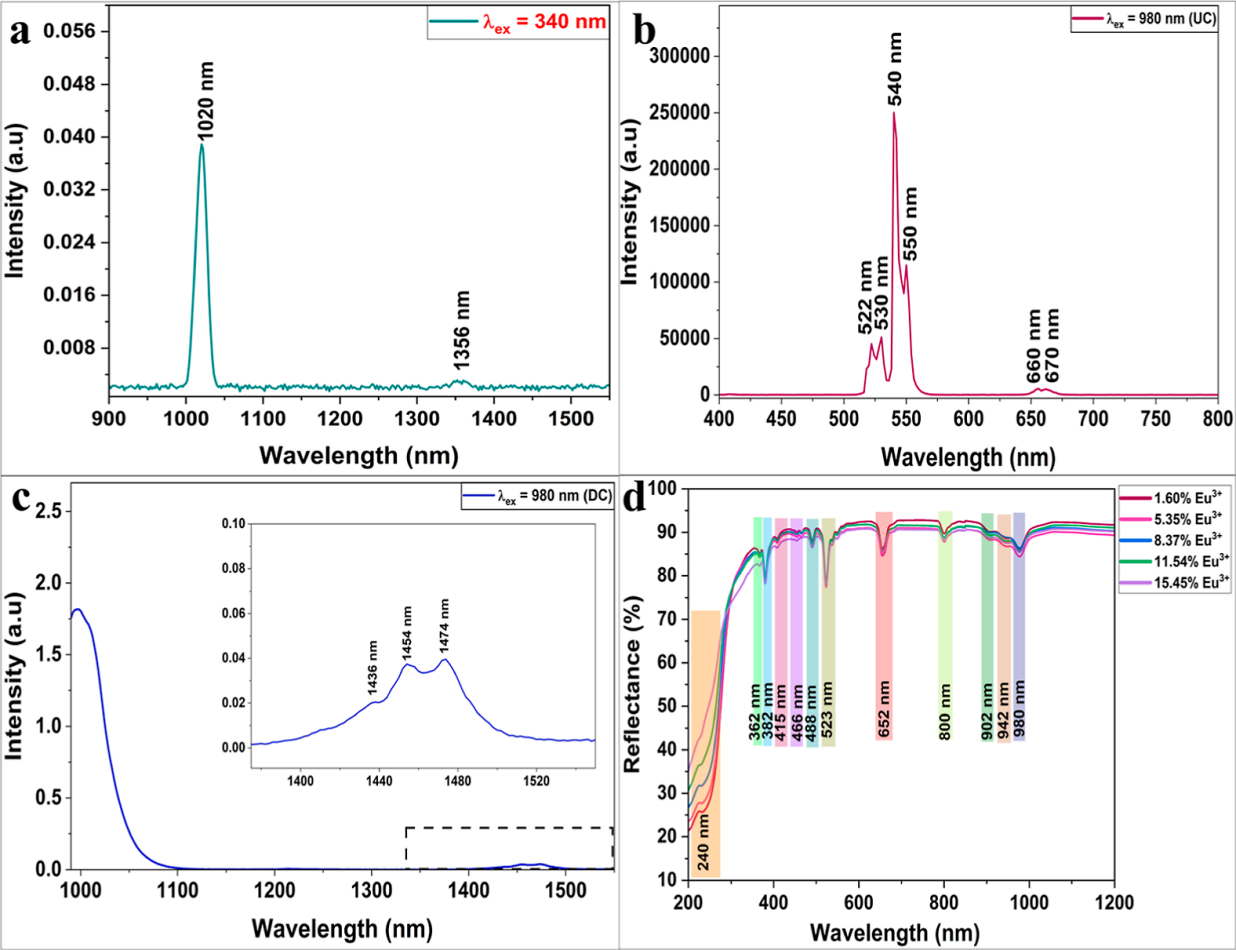
PL spectra of JUP-AS120 at (a) 340 nm excitation. (b) UC and (c) IR emission recorded under 980 nm illumination observed in JUP-AS120. (d) Diffuse reflectance spectra of doped BAO phosphors.

### 3.2 Characterization of Ln^3+^-Doped BAO

Previous reports on the preparation of BAO phosphors as UC materials have utilized dopants such as Er_2_O_3_, Gd_2_O_3_, and Yb_2_O_3_.^30,31^ The UC mechanism typically involves Er^3+^ as the activator and Yb^3+^ as the sensitizer, resulting in visible emissions under 980 nm excitation. Additionally, NIR emissions can be detected using an IR taggant reader. Eu^3+^ is a versatile luminescent ion that plays critical roles in red emission, site symmetry analysis, energy transfer mechanisms, and can serve both as a luminescent probe and as part of dual-valence systems (Eu^3+^/Eu^2+^) for tunable photonic applications. This study demonstrates the incorporation of both Eu^3+^ and Eu^2+^ into the host matrix through ion substitution, despite the absence of a reducing atmosphere.^32,33^

#### 3.2.1 DRS Measurements

The prevalent transitions corresponding to the absorbance region of the phosphors were studied by examining the DRS plots recorded for undoped BAO as well as Ln^3+^ doped BAO in the UV–vis and NIR region. The DRS plot of undoped BAO as presented in Figure S3a shows a broad band ranging between 220 and 270 nm with a peak centered at 240 nm. A similar peak is also observed in doped BAO with different Eu^3+^concentrations, indicating that these transitions belong to the BAO host (Figure 2d). Moreover, several prominent peaks observed at 362, 382, 415, and 466 nm can account for the (^7^F_0_ → ^5^D_4_), (^7^F_0_ → ^5^L_7_), (^7^F_0_ → ^5^D_3_) and (^7^F_0_ → ^5^D_2_) transitions,^34,35^ respectively, observed in Eu^3+^. The free Er^3+^ ion has a 4f^11^ electronic configuration with its ^4^I_15/2_ spin–orbit multiplet ground state resulting in several excited states.^36^ The emergence of sharp peaks at 362 (^4^I_15/2_ → ^4^G_9/2_), 382 (^4^I_15/2_ → ^4^G_11/2_), 488 (^4^I_15/2_ → ^4^F_7/2_), 523 (^4^I_15/2_ → ^2^H_11/2_), 652 (^4^I_15/2_ → ^4^F_9/2_), and 800 nm (^4^I_15/2_ → ^4^I_9/2_) corresponds to intrinsic 4f–4f transitions in the Er^3+^ ion.^36–38^ The peaks at 362 and 382 nm appear collectively from existing transitions in the energy levels of the Eu^3+^ and Er^3+^ ions. The absorbance is stronger at 382, 523, and 652 nm, suggesting the incorporation of both Eu^3+^ and Er^3+^ dopants. The absorbance peaks at 902, 942, and 980 nm are assigned to stark components resulting from excitation of ^2^F_7/2_ (ground state) to ^2^F_5/2_ (excited state).^39^ Evidently, the DRS spectrum indicates the ability of the phosphor to absorb strongly in both visible (523 nm) and NIR (980 nm) regions.

Further, to determine the bandgaps of undoped and doped BAO phosphors, the following Kubelka–Munk equation:^39^ $F\left(R_{\infty }\right)=\frac{K}{S}=\frac{{\left(1-R_{\infty }\right)}^{2}}{2R_{\infty }}$ , where *F*(*R*_∞_) is the Kubelka–Munck function, *K* and *S* are termed as the Kubelka–Munck absorption and scattering coefficients, *R*_∞_ is the reflectance of the sample corresponding to the ideal nonabsorbing standard sample, was used. The direct bandgaps are estimated for undoped and doped BAO phosphors from plot of (*F*(*R*) * *h*υ)^2^ vs energy (*h*υ) by extrapolating the linear component of the curve and listed in Figure S3b–g. BAO with 5.35% Eu^3+^ doping was used for all further studies.

#### 3.2.2 Particle Morphology and Elemental Composition

The SEM images for doped BAO phosphor (Figures S4 and 3a) present particles with a highly porous nature, probably due to the evolution of gases during the synthesis step. Moreover, they display well-defined boundaries. The EDS plot (Figure 3b) indicates the presence of barium (Ba), aluminum (Al), gadolinium (Gd), europium (Eu), oxygen (O), ytterbium (Yb), and erbium (Er) in the doped BAO phosphor.

**Figure 3:**
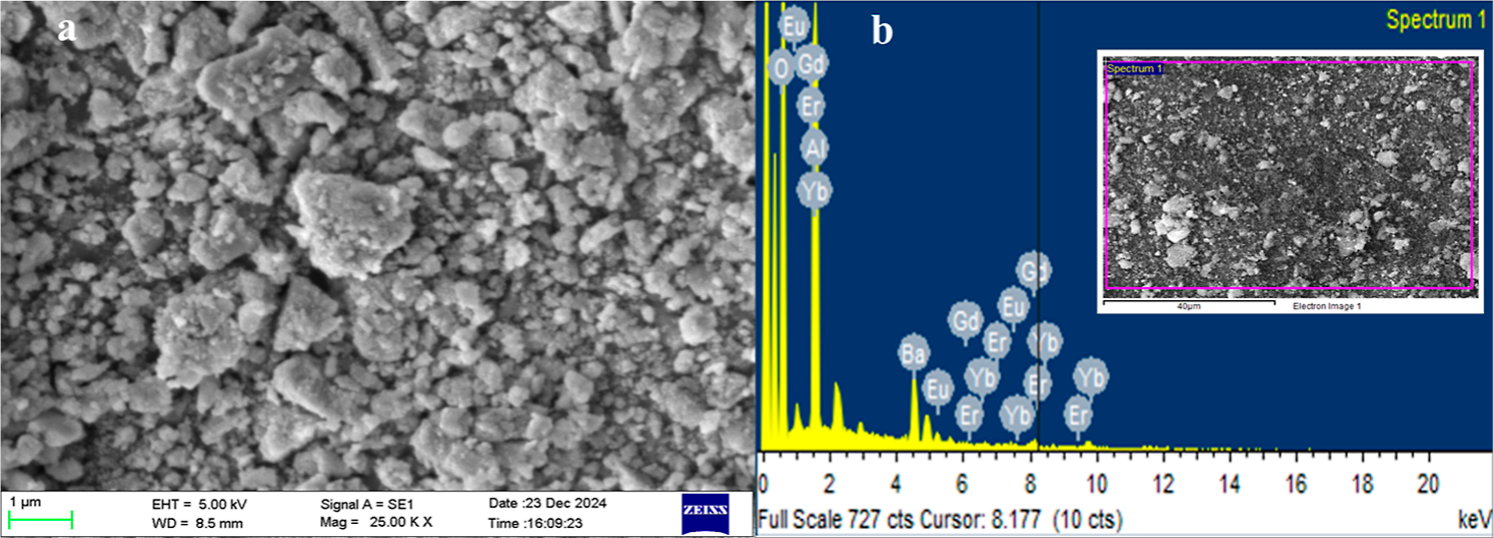
(a) Higher magnification SEM image and (b) EDS plot of the doped BAO phosphor (inset shows the area of interest).

#### 3.2.3 XRD Studies

The XRD pattern obtained for the undoped BAO phosphor synthesized via the solid-state route was systematically compared with the standard diffraction pattern (PDF#72-0387) of BaAl_2_O_4_ in Figure S5. The observed diffraction peaks exhibit excellent agreement with the reference pattern, with no additional peaks corresponding to the secondary phases. This close correspondence confirms the successful formation of phase-pure BAO. The absence of impurity peaks further indicates that the solid-state synthesis is effective in producing a structurally homogeneous BAO phosphor.^40,41^ Furthermore, using Bragg’s equation, the interplanar spacing (*d*-spacing) values for the undoped BAO phosphor were calculated and are listed in Table S2.

XRD patterns for the Eu^3+^-doped BAO phosphors are presented in Figure 4. As outlined in Section 2.1, smaller lanthanide ions such as Gd^3+^, Er^3+^, and Yb^3+^ were initially introduced to promote the substitution of Ba^2+^ sites, thereby improving the lattice accommodation for subsequent Eu^3+^ doping. The introduction of Eu^3+^ resulted in shifts of the diffraction peaks toward higher Bragg angles, which can be attributed to the replacement of the larger Ba^2+^ (*r*_Ba_ ≈ 1.350 Å) ions by the relatively smaller Eu^3+^ (*r*_Eu_ ≈ 0.947 Å) ions within the BAO crystal lattice. This peak shift indicates the reduction in lattice parameters due to the ionic size mismatch and confirms the successful incorporation of Eu^3+^ into the BAO host.^42,43^

**Figure 4:**
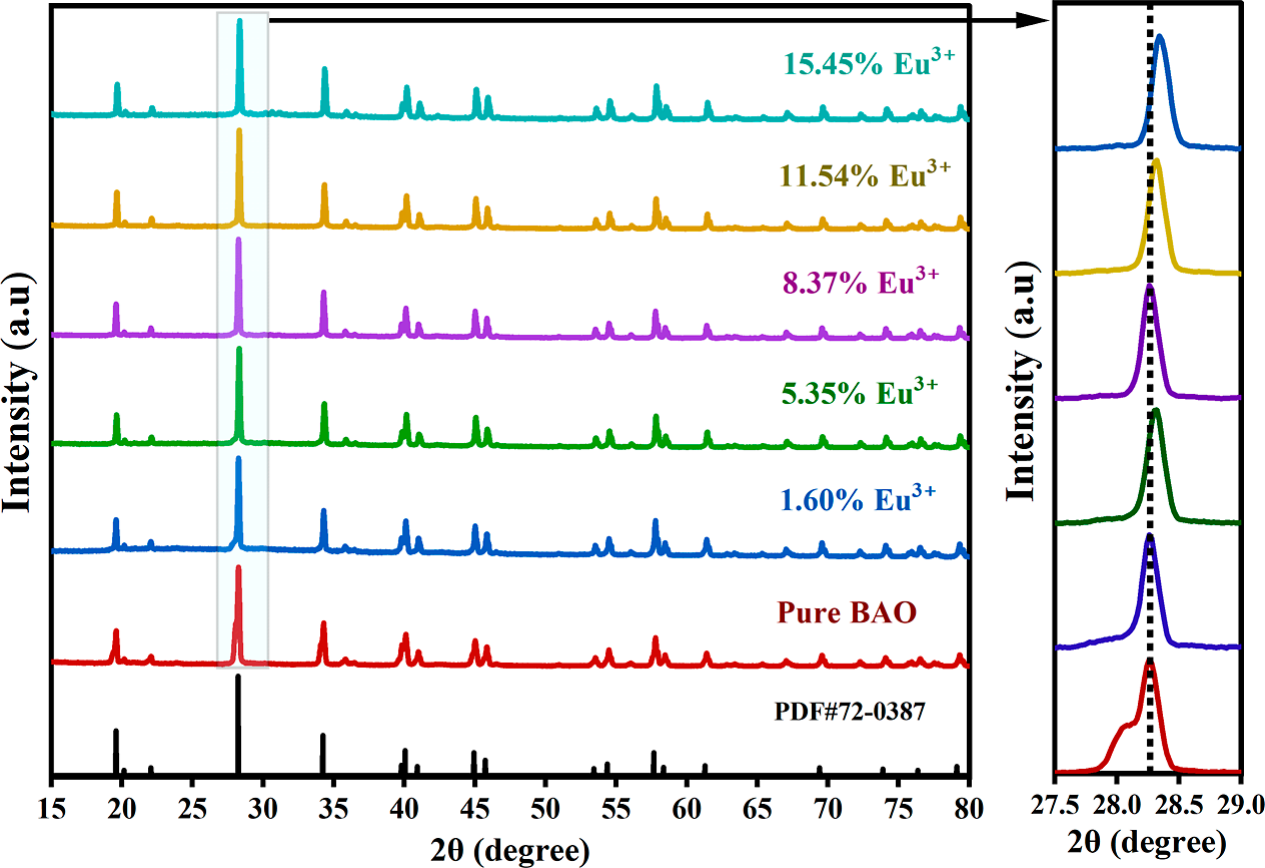
XRD spectra of BAO phosphors with different concentrations of Eu^3+^ doping.

#### 3.2.4 XPS Analysis

The XPS survey scan further confirms the presence of Al, Ba, O, Gd, and Eu in the doped phosphor (Figure 5a) as revealed by the EDS elemental analysis plots. A C 1s peak, likely arising from adventitious carbon contamination, is also observed. Further XPS analysis served in differentiating oxidation states of the Eu element in the BAO matrix. The full scan Eu 3d spectrum (Figure 5b) reveals peaks at 1135 and 1165 eV, corresponding to Eu 3d_5/2_ and Eu 3d_3/2_, respectively, confirming the +3 oxidation state. Additionally, minor peaks at 1125 and 1156 eV indicate the coexistence of Eu^2+^ species in lower concentration.^44,45^ The Ba 3d spectrum shows characteristic doublet peaks at 781 eV (Ba 3d_5/2_) and 796 eV (Ba 3d_3/2_), as presented in Figure S6a. Similarly, the Ba 4d core-level spectrum (Figure S6b) exhibits peaks at 90 eV (Ba 4d_5/2_) and 93 eV (Ba 4d_3/2_). The Al 2p peak at 74 eV is attributed to the Al–O bond, as shown in Figure S6c.^46^ The Gd 3d core-level spectrum (Figure S6d) displays spin–orbit doublet peaks at 1187 and 1219 eV, corresponding to Gd 3d_5/2_ and Gd 3d_3/2_, respectively, along with a peak at 136 and 142 eV (Gd 4d_5/2_ and Gd 4d_3/2_) in Figure S6e.^47,48^ The corresponding satellite peaks for Gd 3d core spectrum (Figure S6d) are also identified at 1197, 1229, and 1236 eV. This indicates the +3 oxidation state of Gd and its successful integration into the BAO matrix. The 4d doublet for Er appears at 168 (Er 4d_5/2_) and 171 eV (Er 4d_3/2_) for Er (Figure S6f) while for Yb the 4d core level (Figure S6g) peaks are observed at 186 (Yb 4d_5/2_) and 190 eV (Yb 4d_3/2_). This indicates the successful incorporation of Er^3+^ and Yb^3+^ in the BAO host.^47,49^

**Figure 5:**
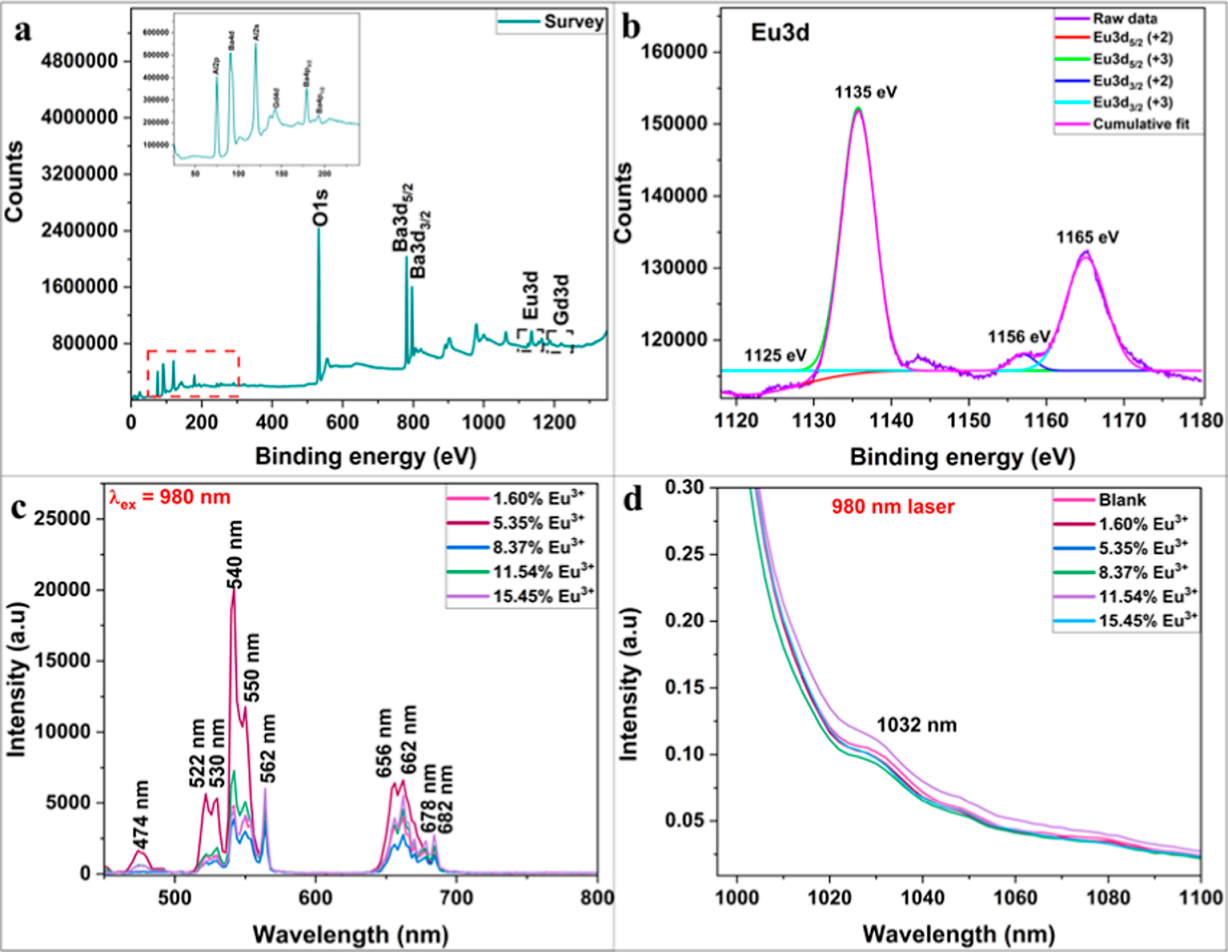
(a) Survey scan and (b) full scan XPS spectra depicting coexistence of both Eu^3+^ and Eu^2+^ oxidation states. PL spectra of doped BAO recorded in the (c) visible and (d) NIR regions upon 980 nm excitation.

#### 3.2.5 Emission Studies

Photoluminescence (PL) studies were conducted to investigate the luminescence behavior of Gd^3+^ and Er^3+^ and to confirm the partial reduction of Eu^3+^ in the doped BAO phosphor. Emission peaks at 300 and 312 nm, recorded under excitation at 274 nm, correspond to the ^6^P_5/2_ → ^8^S_7/2_ and ^6^P_7/2_ → ^8^S_7/2_ transitions of Gd^3+^ ions, respectively (Figure S7b,c).^50,51^ Upon excitation at 264, 274, 285, and 312 nm, a broad emission band spanning the UV to green region (380–550 nm) is observed (Figure S7a,b,d,f). Among these, a prominent emission peak at 466 nm is attributed to the 4f^6^5d^1^ → 4f^7^ transition^46^ of Eu^2+^. Additional emissions in the range of 504–517 nm are detected under excitations at 370, 377, 382, and 396 nm (Figure S7h–k), also corresponding to allowed transitions^52,53^ of Eu^2+^. Notably, under 396 nm excitation (Figure S7k), characteristic emissions at 650 nm (^5^D_0_ → ^7^F_3_), and 700 nm (^5^D_0_ → ^7^F_4_) are observed. These peaks are ascribed to the 4f–4f forbidden transitions of Eu^3+^, indicating its partial reduction to the Eu^2+^ state.^54,55^ The Er^3+^ ions exhibit spectral features under various excitations: 362 nm (^4^I_15/2_ → ^4^G_9/2_), 382 nm (^4^I_15/2_ → ^4^G_11/2_), 450 nm (^4^I_15/2_ → ^4^F_5/2_), as well as 466 and 481 nm (^4^I_15/2_ → ^4^F_7/2_) (Figure S7g,j,l–n).^30,40^ Emissions observed at 511–528 nm, 540 nm, and 630–665 nm are attributed to ^2^H_11/2_ → ^4^I_15/2_, ^4^S_3/2_ → ^4^I_15/2_ and ^4^F_9/2_ → ^4^I_15/2_ transitions, respectively.^56^

Phosphors exhibiting UC phenomenon typically comprise Yb^3+^ as sensitizers coupled with Er^3+^ as activators. Upon excitation at 980 nm, Er^3+^ ions are promoted to intermediate energy levels corresponding to the ^4^I_15/2_ → ^4^I_11/2_ transition. Yb^3+^ having a higher absorption cross section at 980 nm are excited to higher energy level (^2^F_7/2_ → ^2^F_5/2_). Due to dipole–dipole interactions, energy is transferred from Yb^3+^ to Er^3+^, further exciting the Er^3+^ ions to higher energy states. The characteristic emissions observed in the visible region arise from the intra4f transitions of the Er^3+^ ions. Figure 5c demonstrates the UC emissions recorded for doped BAO. The peaks at 474, 522–530 and 540–562 nm can be assigned to ^2^H_9/2_ → ^4^I_15/2_, ^2^H_11/2_ → ^4^I_15/2_ and ^4^S_3/2_ → ^4^I_15/2_ transitions,^57^ while the less intense emissions at 662–678 nm correspond to ^4^F_9/2_ → ^4^I_15/2_ transition. Er^3+^ also exhibits an NIR emission for excitation at 980 nm (Figure 5d). Among the different doped BAOs prepared, the phosphor with 5.35% Eu^3+^ demonstrated an intense green emission (542 nm) in addition to a minor red emission (662 nm).

The UC phenomenon is achieved by the absorption of two photons, thereby populating the energy levels in the activator ion (Er^3+^). The first process is excited state absorption involving continuous absorption of two photons followed by energy transfer UC mechanism commonly observed in sensitizer-activator coupled systems. Photon avalanche UC demonstrates nonlinear increase in luminescence with subsequent increase in excitation power. The first report on photon avalanche was in Pr^3+^ doped LaCl_3_ and LaBr_3_ crystals. Moreover, the accounts on photon avalanche are reported in single doped (Er^3+^, Tm^3+^, Ho^3+^, Pr^3+^) or double doped lanthanides (Ho^3+^/ Yb^3+^, Er^3+^/Yb^3+^, Ho^3+^/Tm^3+^).^58,59^ Generally, the number of photons accompanied during the photon avalanche process is greater than the normal UC process. The underlying mechanism was comprehended by measuring the emissions under various 980 nm excitation power as portrayed in Figure 6a–e. The minimum laser power of 350 mW was used to record the spectral peaks, and a further increase in excitation power resulted in nonlinear increase in emission intensities (Figure S8a–e) mainly for 562 nm (for 1.60%, 8.37%, 11.54% and 15.45% Eu^3+^ concentrations) and 542 nm (for 5.35% Eu^3+^ concentration) unlike the linear increment observed in normal UC process.^60,61^ Interestingly, there was a linear increase in intensities (for 542 and 562 nm) up to 440 mW laser excitation and further an exponential increase indicating enhanced population in higher (^4^F_7/2_) levels. The shift toward lower levels ^2^H_11/2_ levels due to nonradiative transition results in prominent green emission. The number of photons can be estimated using the expression I α P^n^, where I is the variation in emission intensity as a function of laser power excitation (P) and n is the number of photons calculated estimated from the slope obtained by applying a linear fit to the log of emission intensities as a function of laser excitation power. The fitting of logarithmic power-pump dependency on emission intensities (Figures S9–S13) calculated for doped BAO phosphors with different Eu^3+^ concentrations indicates the participation of two or more photons during photon avalanche UC process. The greater value of n corresponding to lower laser power further indicates the photon avalanche process.^62^

**Figure 6:**
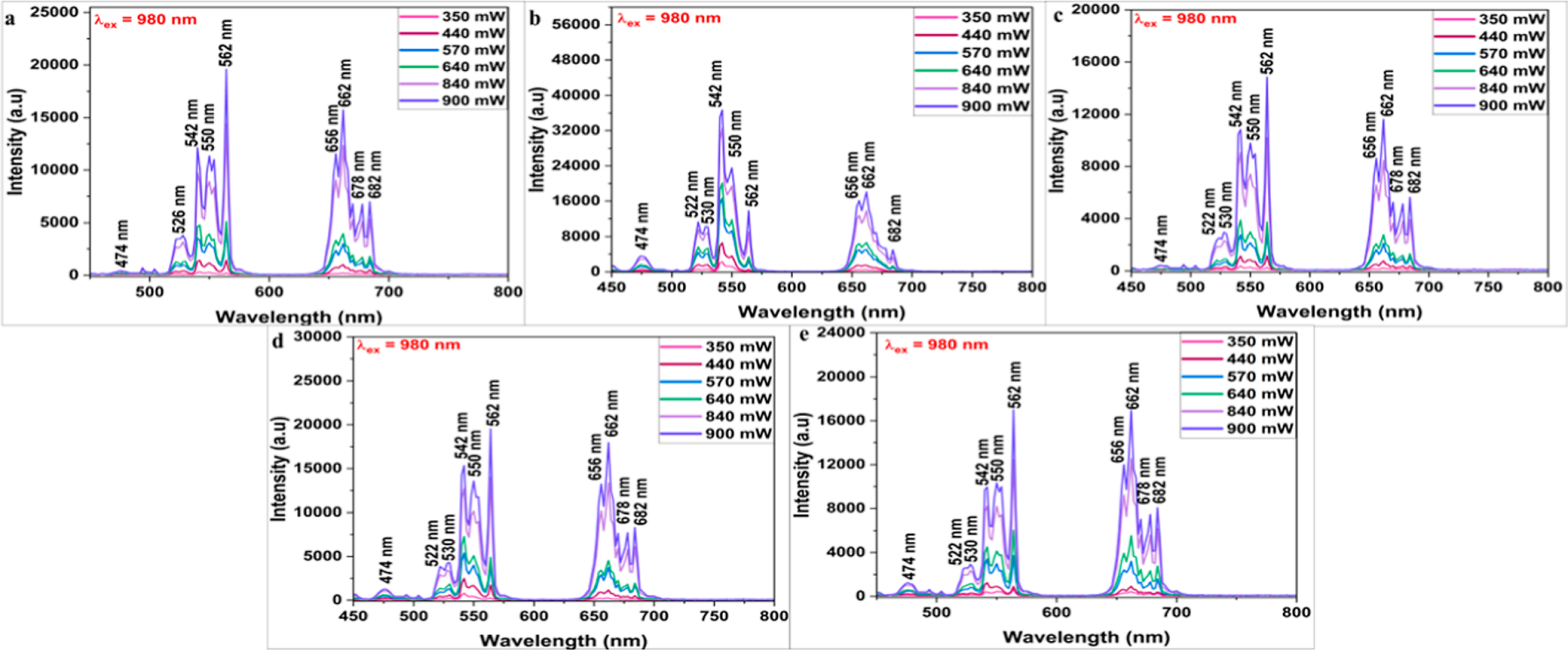
UC emission recorded using different 980 nm laser power (350, 440, 570, 640, 840, and 900 mW) for (a) 1.60%, (b) 5.35%, (c) 8.37%, (d) 11.54%, and (e) 15.45% Eu^3+^-doped BAO phosphors.

### 3.3 Study of Properties of Secure and Multisecure Adhesives

The JUP-AS120 powder was infused to Swifttherm 8440 to obtain a secure adhesive, whereas the composite powder of 5.35% Eu^3+^-doped BAO and JUP-AS120 was infused to Swifttherm 8440 to prepare a multisecure adhesive. The newly prepared glues were faintly milky white in color compared to Swifttherm 8440 (control adhesive) as shown in Figure 7a–c.

**Figure 7:**
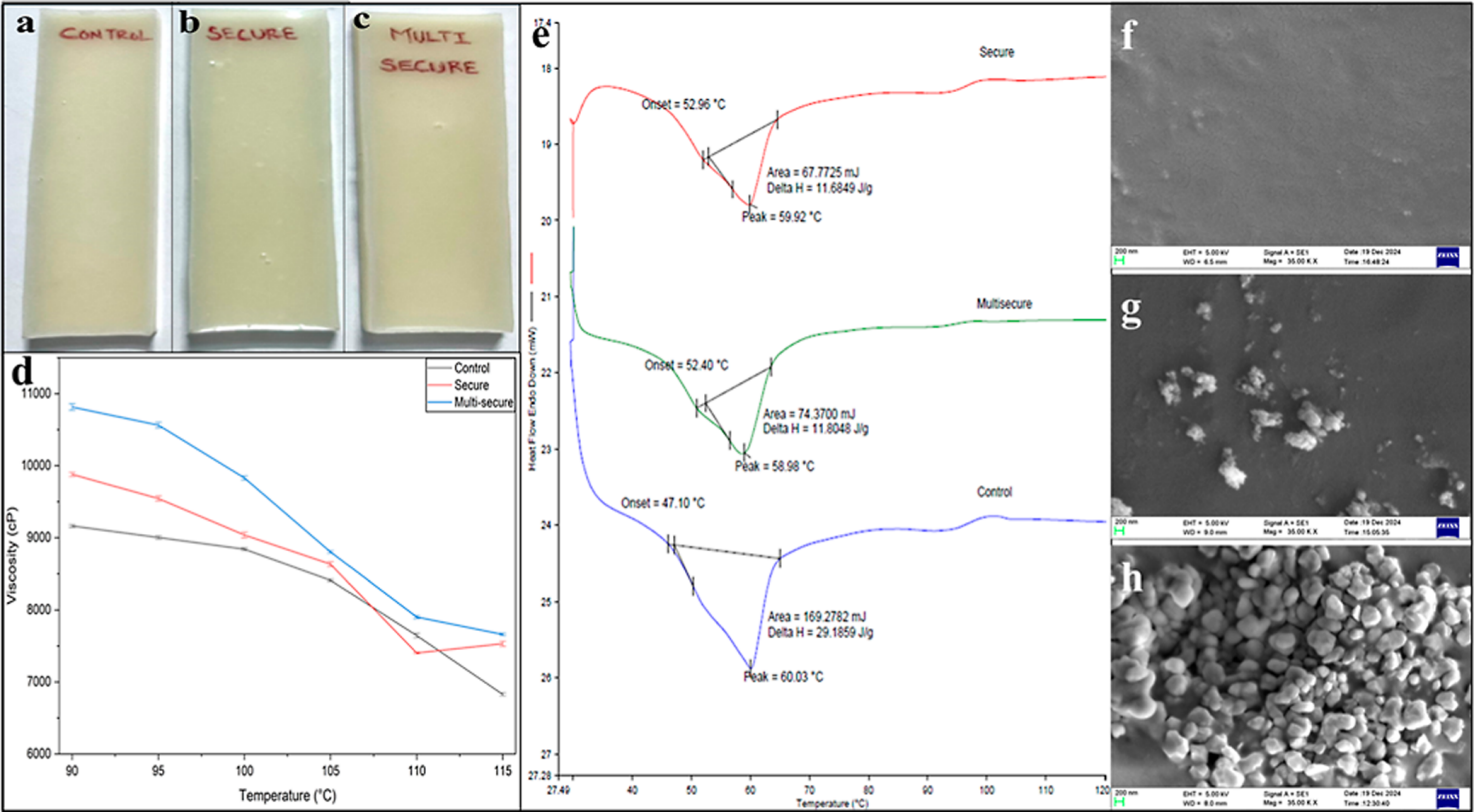
Photographs of (a) control, (b) secure, and (c) multisecure adhesives under daylight. (d)­Temperature-dependent viscosity variations for control, secure, and multisecure adhesives. (e) DSC thermograms of control, secure, and multisecure adhesives. SEM images of (f) control, (g) secure, and (h) multisecure adhesives.

#### 3.3.1 Viscosity Studies

The control, secure, and multisecure adhesives that are solids at ambient conditions were melted to study the viscosities at various temperatures and the results are listed in Table S3. The graphical representation for temperature-dependent viscosity measurements for secure and multisecure adhesives shows only marginal changes in the viscosity values compared to the control at lower temperatures studied from 90 to 115 °C as visualized in Figure 7d. At higher temperatures, adhesives generally exhibit lower viscosity due to lower cohesive forces, allowing their easy flow. The viscosity values of all three adhesives show minimal variation at elevated temperatures, which confirms the practical applicability of the two modified adhesives as replacements for the control adhesive.

#### 3.3.2 Thermal Studies

To study the impact of infusion of JUP-AS120 and the composite phosphor on the thermal properties of Swifttherm 8440, DSC studies were performed. The DSC thermograms (Figure 7e) of control, secure, and multisecure adhesives exhibited their respective melting at 60.03, 59.92, and 58.98 °C, which suggests that the incorporation of the phosphor/s did not affect the melting temperature of the control adhesives significantly.

#### 3.3.3 Morphological Studies

The SEM microimage acquired for the control adhesive shows a smooth surface (Figure 7f). The secure adhesive displays amorphous particles with a wide variety of morphologies (Figure 7g), whereas the SEM image of multisecure adhesive exhibits denser particle distribution (Figure 7h) as it incorporates both the phosphors. Notably, the multisecure adhesive has a greater pigment load in comparison to the secure adhesive.

#### 3.3.4 Emission Studies

The emission profiles of the control, secure, and multisecure glues were assessed at both 340 and 980 nm excitations. The secure adhesive shows a greenish-yellow fluorescence (λ_em_ = 524–554 nm), whereas the multisecure glue displays a green fluorescence (λ_em_ = 540 nm) under a 980 nm light source as presented in Figure 8a. In addition, secure and multisecure adhesives display comparatively weak red emissions at 658–670 nm and 650–662 nm, respectively. Thus, the use of a green emission cutoff filter or a 610 nm band-pass filter enables us to view exclusively the red fluorescence from the modified adhesives (Figure 8b). The control adhesive does not demonstrate any remarkable changes in fluorescence under 980 nm without and with a green emission cutoff filter (Figure 8a,b). Upon UV excitation at 340 nm and IR excitation at 980 nm, the control glue did not show any emission, whereas both secure and multisecure adhesive samples displayed IR emissions attributed to the phosphors as portrayed in Figure S14a,b. The color coordinates for secure (0.2905, 0.6898) and multisecure (0.2468, 0.7312) adhesives upon illumination with 980 nm correspond to the green region in the absence of 610 nm band-pass filter as depicted in Figure 8c,e. The inset images show yellowish-green and bright green fluorescence from secure and multisecure glues. While under the same light source and in the presence of a green cutoff filter, the color coordinates for secure (0.7245, 0.2751) and multisecure (0.7114, 0.2877) glues, respectively, are as shown in Figure 8d,f. The red fluorescence from secure and pink fluorescence from multisecure adhesives can be visualized in the inset of these figures.

**Figure 8:**
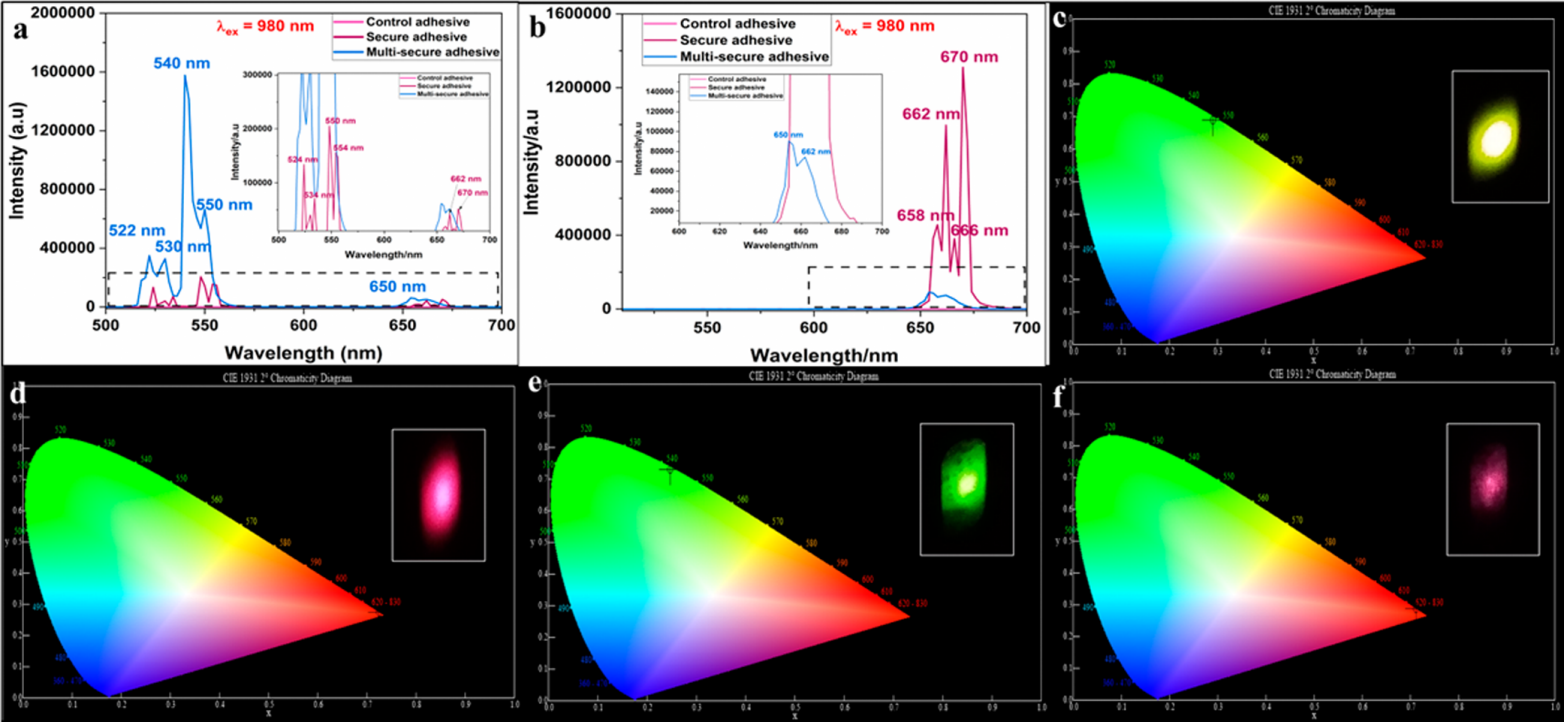
PL spectra for the adhesives in the (a) absence and (b) presence of a 610 nm band-pass filter under 980 nm excitation. CIE plot for (c) secure and (e) multisecure adhesives in the absence of a 610 nm band-pass filter (inset: photograph of the yellowish-green and bright green fluorescence). CIE plot for (d) secure and (f) multisecure adhesives in the presence of a 610 nm band-pass filter (inset: photograph of the red and pink fluorescence); λ_ex_ = 980 nm.

#### 3.3.5 Photostability Assessment

It is crucial to assess the photobleaching effect of the two different phosphors incorporated in the multisecure adhesive for real-time applications. Therefore, a photostability study was performed by attaching a small portion of the multisecure adhesive to a metal plate and subjecting to light fastness test. The sample was exposed to 10, 20, and 30 cycles at a speed of 30 m/min under 300 W of UV irradiation. The PL spectra (Figure S15a) of the UV-exposed samples reveal an initial decline in intensity after 10 cycles, while further prolonged exposures show no significant impact on the overall photostability of the adhesive under UV light.

### 3.4 Application of Adhesives to the Spine of a Book

#### 3.4.1 Security Features for Real-Life Applications

Book counterfeiting often goes undetected, with duplicated copies being rebranded and sold in the market, resulting in significant loss of revenue for the original publisher or author. To address this, we demonstrate the application of the prepared security glue in book binding as a real-world example, incorporating authentication features that allow originality verification of the book and serve in combating its unauthorized duplication. The modified adhesive was applied to the spine of a book made from maplitho paper stock. Under daylight conditions, the area (a window created to expose the glued area in this case) with the secure adhesive appears similar to any standard adhesive (Figure 9a). When illuminated using a UV source (λ_ex_ = 365 nm) the adhesive demonstrates blue fluorescence (λ_em_ = 450 nm), which serves to initially deceive the counterfeiter regarding its embedded security features (Figure S15b). However, when illuminated with a 980 nm light source, bright green and red fluorescence in the absence and presence of a 610-band-pass filter as presented in Figure 9b,c, respectively, is observed. Additionally, scanning the adhesive-applied area of the book spine with a commercially available 940–980 nm IR taggant (InfraRead 601) device produces a beep sound, as shown in the Supporting Information Video. The device consists of a laser diode (λ_ex_ = 980 nm) source, photodetector, IR light-emitting diodes (LEDs), and a beeper. The photodetector is receptive to IR emissions generated when the 980 nm light source is incident on the modified adhesives. This triggers response toward onboard processor to send a signal to the beeper and IR LED to induce audio and blinking responses from the device. Thus, the infusion of security markers into the glue system facilitates high protection to the product, which cannot be easily forged. The multilevel security features that include both optical and audio authentication enable easy validation of the product by the user.

**Figure 9:**
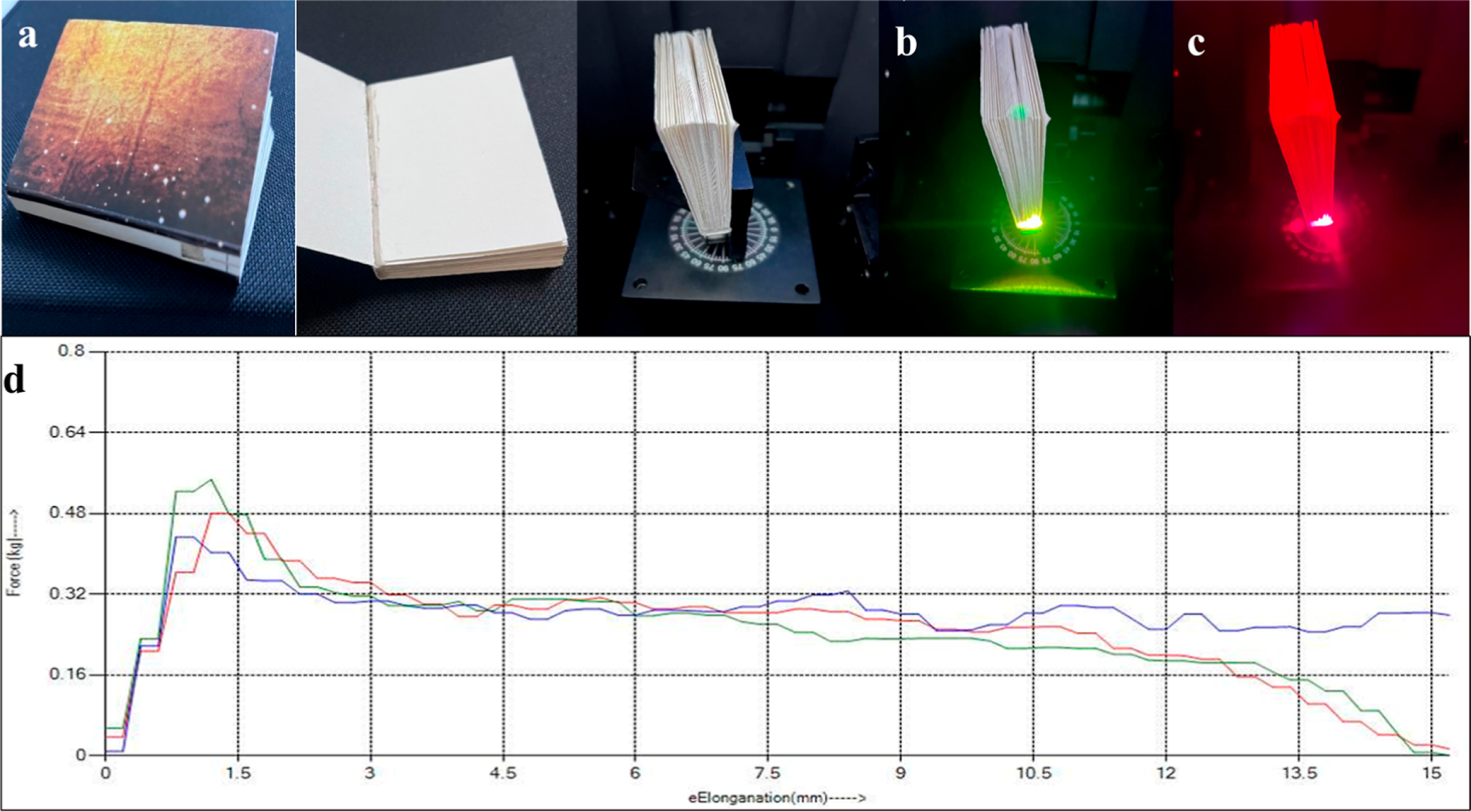
Photographs of the spine of the book glued using a modified adhesive under (a) daylight and under a 980 nm laser (b) without and (c) with a 610 nm band-pass filter. (d) Peel strength test for control (red line), secure (blue line), and multisecure (green line) adhesives measured at varying applied forces.

#### 3.4.2 Adherence Test

Peel or seal testing is performed on the adhesives to evaluate peel separation strength of the adhesive laminated paper sample. A similar peel test was performed on the control, secure, and multisecure adhesives under varying applied forces and the respective plots are shown in Figure 9d. The control adhesive shows a maximum peel strength at 0.48 kgf, beyond which it tears off. Interestingly, the secure adhesive exhibits a peel strength of 0.43 kgf, while the multisecure adhesive demonstrates a maximum peel strength of 0.55 kgf. Thus, the incorporation of the phosphors did not affect the adherence of the adhesives compared with the control. Moreover, the multisecure glue shows slightly enhanced resistance to peeling.

## 4 Conclusion

Conventional anticounterfeiting techniques, such as barcodes, QR codes, or visible special markings, are often vulnerable to tampering, removal, or replication. The present work reports the incorporation of UC emissive pigments directly into adhesives commonly used in book binding applications. The aim is to introduce a concealed, material-level security feature that is difficult to replicate or remove without compromising the book’s integrity and facilitate the identification of counterfeit books that lack established security features. As the adhesive is integral to the binding process; any modifications remain invisible externally but can be authenticated through multimodal responses. When exposed to a 980 nm NIR light, the adhesive exhibits a green fluorescence. Using a green emission cutoff filter allows the red emission to be visualized as an additional optical signal. Furthermore, an audio response is triggered via an IR taggant reader, providing another layer of authentication. Thus, the modified adhesives offer dual-mode security: visual fluorescence and audio feedback, under 980 nm excitation and using IR detector, respectively, enabling a multilevel anticounterfeiting system. This technique of embedding security features provides an additional layer of protection tailored to counter increasingly sophisticated counterfeiting methods, thereby complementing rather than replacing conventional overt measures, and can potentially be adapted for use with other compatible adhesives employed in various industrial applications.

## Supplementary Material





## Data Availability

Supplementary data and video provided. All data reported in this manuscript are available on request.

## Notes

Survey spectrum and core spectra for JUP-AS120; XRD spectrum of the JUP-AS120 pigment; DRS spectrum for the undoped BAO phosphor; direct bandgaps of undoped and doped BAO phosphors; lower magnification SEM image of the doped BAO phosphor; XRD spectra of the undoped BAO phosphor overlaid with reference patterns of BaAl_2_O_4_ (PDF#72-0387); full scan spectra of Ba 3d, Ba 4d, Al 2p, Gd 3d, Gd 4d, Er 4d and Yb 4d & Ba 4p; PL spectra of various Eu^3+^-doped BAO phosphors; variation of UC intensities of 474, 522, 530, 542, 550, 562, 656, 662, and 682 nm emission bands with increasing laser power for doped phosphors; logarithmic power-pump dependency on 474, 522, 530, 542, 550, 562, 656, 662, and 682 nm emission intensities for Eu^3+^-doped BAO phosphors under a 980 nm excitation; NIR emissions of adhesives recorded at 340 and 980 nm excitations; PL spectra of a multisecure adhesive sample exposed to different UV passage cycles; PL spectra for a multisecure adhesive at a 365 nm excitation; *D*-space value estimated using Bragg’s equation for JUP-AS120 and BAO; and temperature-dependent viscosity values for control, secure, and multisecure adhesives (PDF)
Demonstration of security features of the adhesive applied to the spine of a book for a real-life application (MP4)
