# Supplementary material for: Smart Adhesives with Multilevel Security Features for Real-World Anticounterfeiting Applications
Source: ACS Omega. 2025 Dec 31;11(1):877–88. doi: 10.1021/acsomega.5c07717 (PMC12809865; doi:10.1021/acsomega.5c07717)
Supplement: Supplementary file 1 [file ao5c07717_si_001.pdf]

Smart adhesives with multi-level security features for real-world anti-counterfeiting applications

Anand P J[a,b], Namratha Ullal[b], Dhanya Sunil[b*], Kiran R[c], Nagabhushana Nayak[a], Ashok Rao[c]

[a]Manipal Technologies Limited, Manipal 576104, Karnataka, India.

[b]Department of Chemistry, Manipal Institute of Technology, Manipal Academy of Higher Education, Manipal 576104, Karnataka, India.

[c]Department of Physics, Manipal Institute of Technology, Manipal Academy of Higher Education, Manipal 576104, Karnataka, India.

SUPPLEMENTARY FIGURES

1. **Figure S1:** a) Survey spectrum and core spectra of b) Na 1s, c) Y 3d, d) F 1s, e) Yb 4d and f) Er 4d for JUP-AS120.
2. **Figure S2:** XRD spectrum of the JUP-AS120 pigment.
3. **Figure S3:** a) DRS spectrum for undoped BAO phosphor. Direct bandgaps of b) undoped and c) 1.60 %, d) 5.35 %, e) 8.37 %, f) 11.54 % & g) 15.45 % Eu^{3+} doped BAO phosphors.
4. **Figure S4:** Lower magnification SEM image of doped BAO phosphor.
5. **Figure S5:** XRD spectra of undoped BAO phosphor overlaid with reference patterns of $BaAl_2O_4$ (PDF#72-0387).
6. **Figure S6:** Full scan spectra of a) Ba3d, b) Ba4d, c) Al2p, d) Gd3d, e) Gd4d, f) Er4d and g) Yb4d & Ba4p.
7. **Figure S7:** Photoluminescence spectra of various Eu^{3+} doped BAO phosphors recorded at a) 264 nm, b) - c) 274 nm with and without 370 nm optical filter, d) - e) 285 nm with and without 370 nm optical filter, f) 312 nm, g) 362 nm, h) 370 nm, i) 377 nm, j) 382 nm, k) 396 nm, l) 450 nm, m) 466 nm and n) 481 nm.
8. **Figure S8:** Variation of UC intensities of 474, 522, 530, 542, 550, 562, 656, 662 and 682 nm emission bands with increasing laser power for a) 1.60% Eu^{3+}, b) 5.35% Eu^{3+}, c) 8.37% Eu^{3+}, d) 11.54% Eu^{3+} and e) 15.45% Eu^{3+} doped phosphors.
9. **Figure S9:** The logarithmic power-pump dependency on a) 474, b) 522, c) 530, d) 542, e) 550, f) 562, g) 656, h) 662 and i) 682 nm emission intensities for 1.60% Eu^{3+} doped BAO under 980 nm excitation.
10. **Figure S10:** The logarithmic power-pump dependency on a) 474, b) 522, c) 530, d) 542, e) 550, f) 562, g) 656, h) 662 and i) 682 nm emission intensities for 5.35 % Eu^{3+} doped BAO under 980 nm excitation.

11. **Figure S11:** The logarithmic power-pump dependency on a) 474, b) 522, c) 530, d) 542, e) 550, f) 562, g) 656 h) 662 and i) 682 nm emission intensities for 8.37 % Eu^{3+} doped BAO under 980 nm excitation.
12. **Figure S12:** The logarithmic power-pump dependency on a) 474, b) 522, c) 530, d) 542, e) 550, f) 562, g) 656 h) 662 and i) 682 nm emission intensities for 11.54 % Eu^{3+} doped BAO under 980 nm excitation.
13. **Figure S13:** The logarithmic power-pump dependency on a) 474, b) 522, c) 530, d) 542, e) 550, f) 562, g) 656 h) 662 and i) 682 nm emission intensities for 15.45 % Eu^{3+} doped BAO under 980 nm excitation.
14. **Figure S14:** NIR emissions of adhesives recorded at a) 340 and b) 980 nm excitations.
15. **Figure S15:** a) PL spectra of multi-secure adhesive sample exposed to different UV passage cycles. b) PL spectra for multi-secure adhesive at 365 nm excitation (inset image consisting multi-secure adhesive under 365 nm UV light).

SUPPLEMETARY TABLES

1. **Table S1:** D-space value estimated using Bragg's equation for JUP-AS120.
2. **Table S2:** D-space value estimated using Bragg's equation for BAO.
3. **Table S3:** Temperature dependent viscosity values for control, secure and multi-secure adhesives.

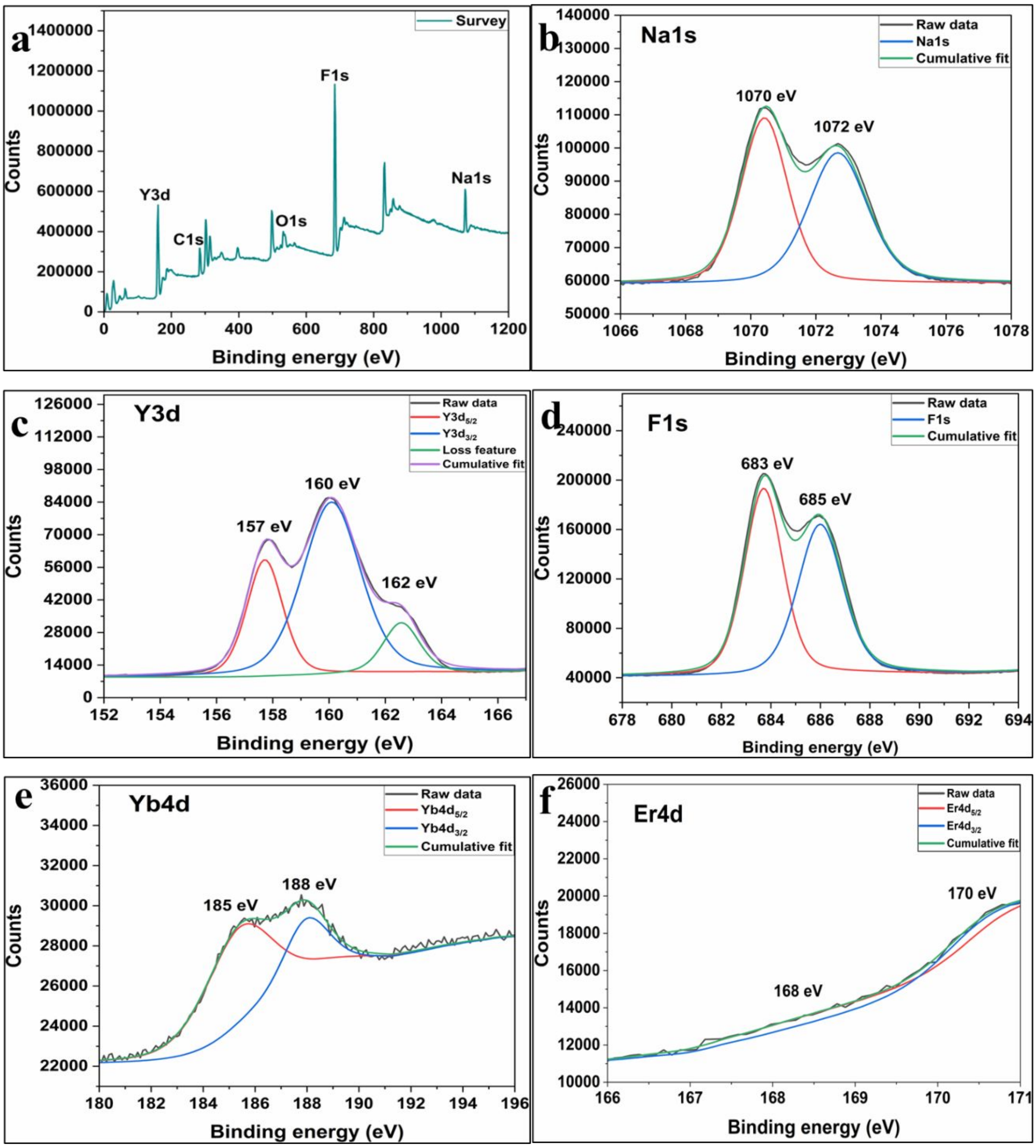

Figure S1: a) Survey spectrum and core spectra of b) Na 1s, c) Y 3d, d) F 1s, e) Yb 4d and f) Er 4d for JUP-AS120.

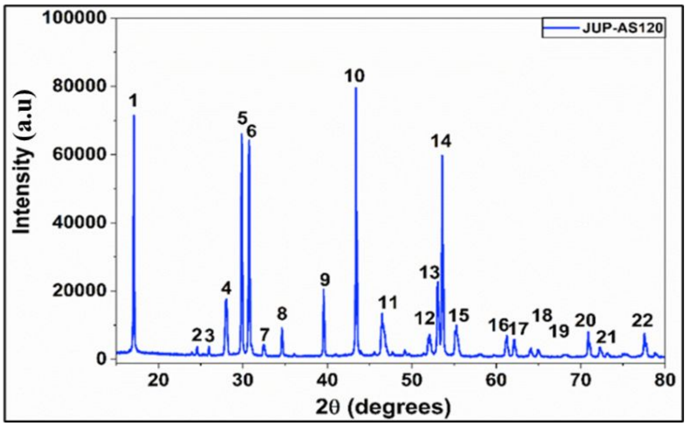

Figure S2: XRD spectrum of the JUP-AS120 pigment.

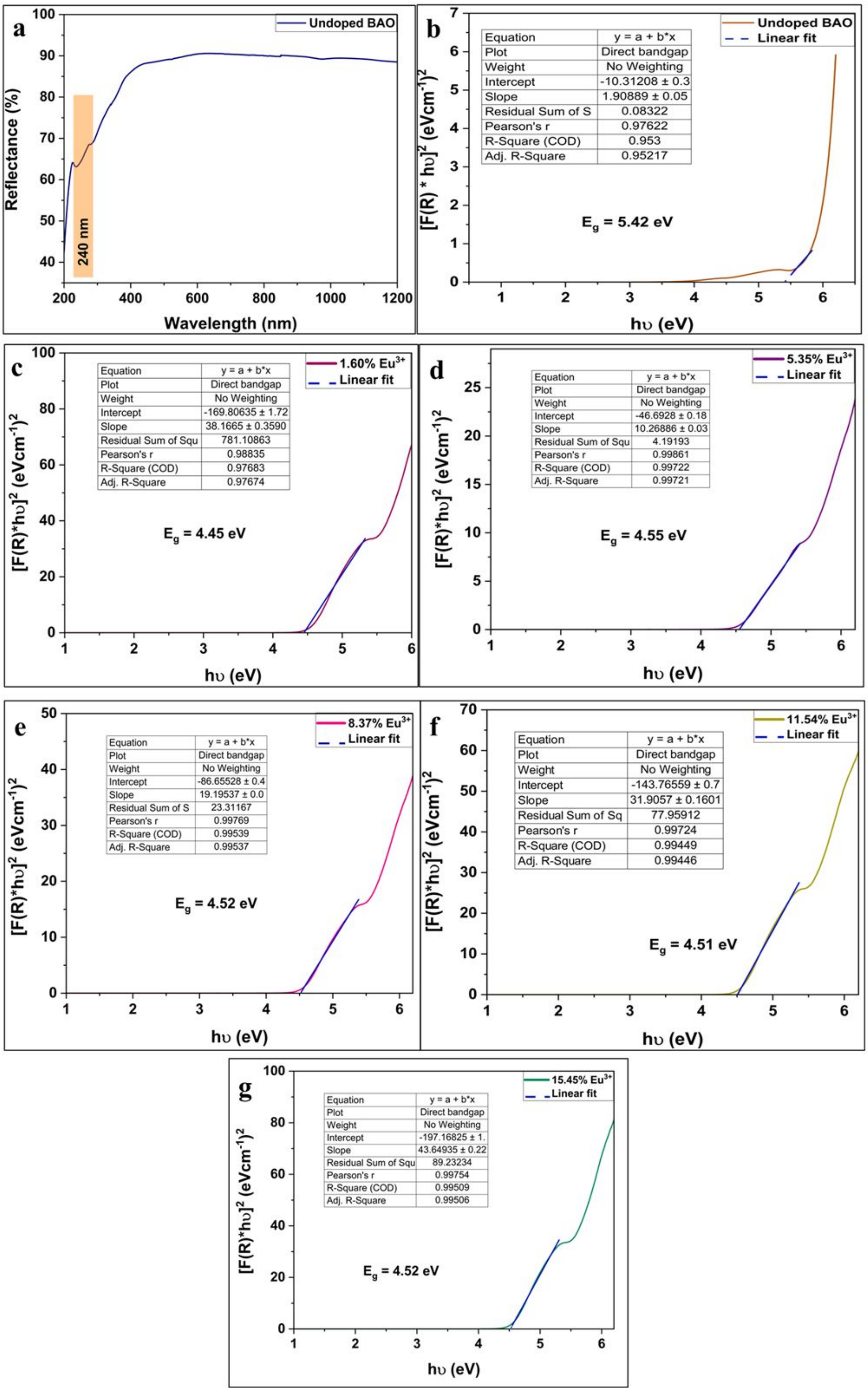

Figure S3: a) DRS spectrum for undoped BAO phosphor. Direct bandgaps of b) undoped and c) 1.60 %, d) 5.35 %, e) 8.37 %, f) 11.54 % & g) 15.45 % Eu^{3+} doped BAO phosphors.

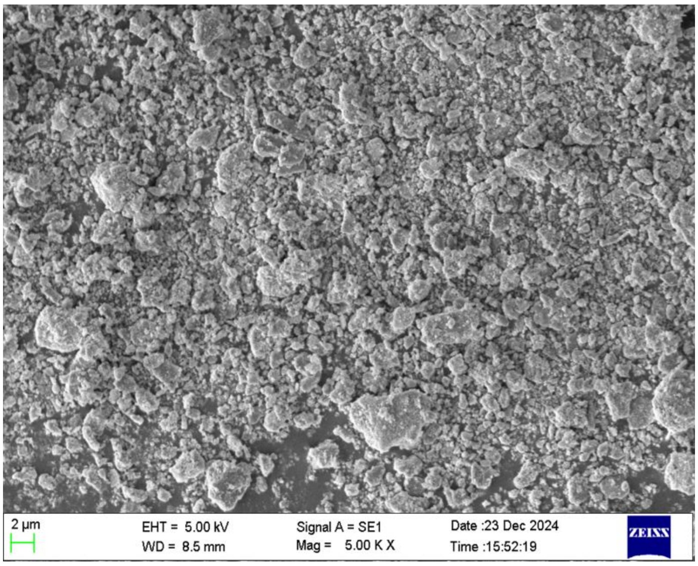

Figure S4: Lower magnification SEM image of doped BAO phosphor.

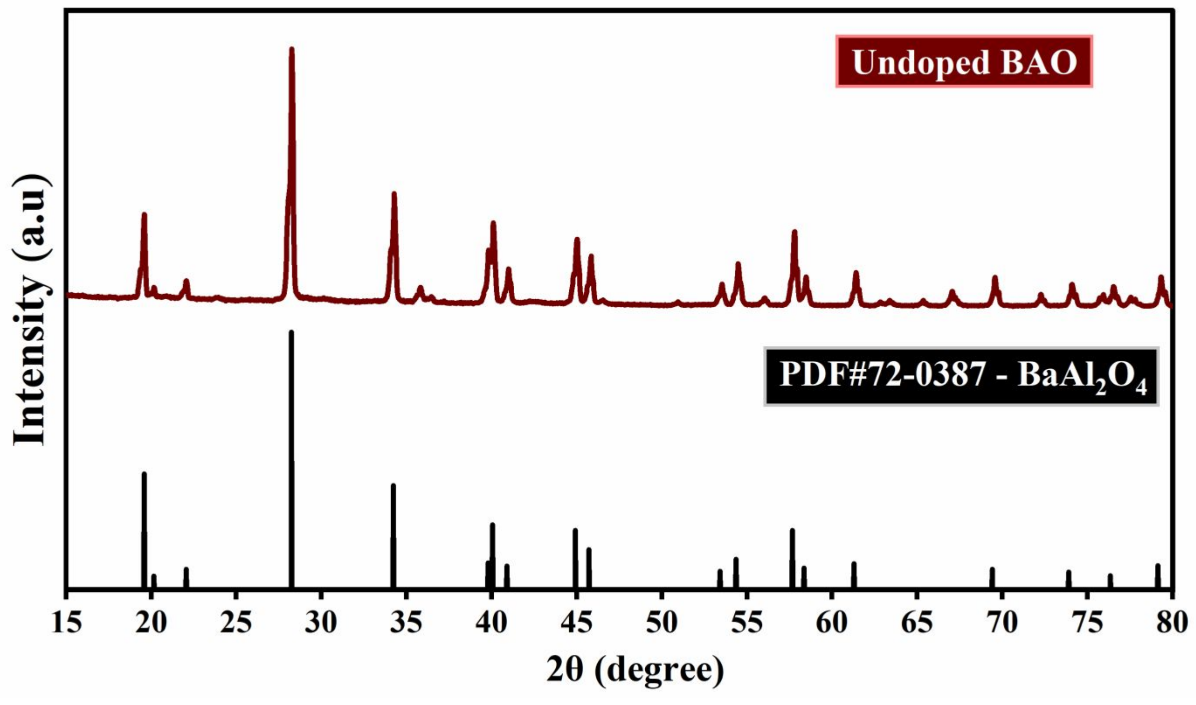

Figure S5: XRD spectra of undoped BAO phosphor overlaid with reference patterns of $BaAl_2O_4$ (PDF#72-0387).

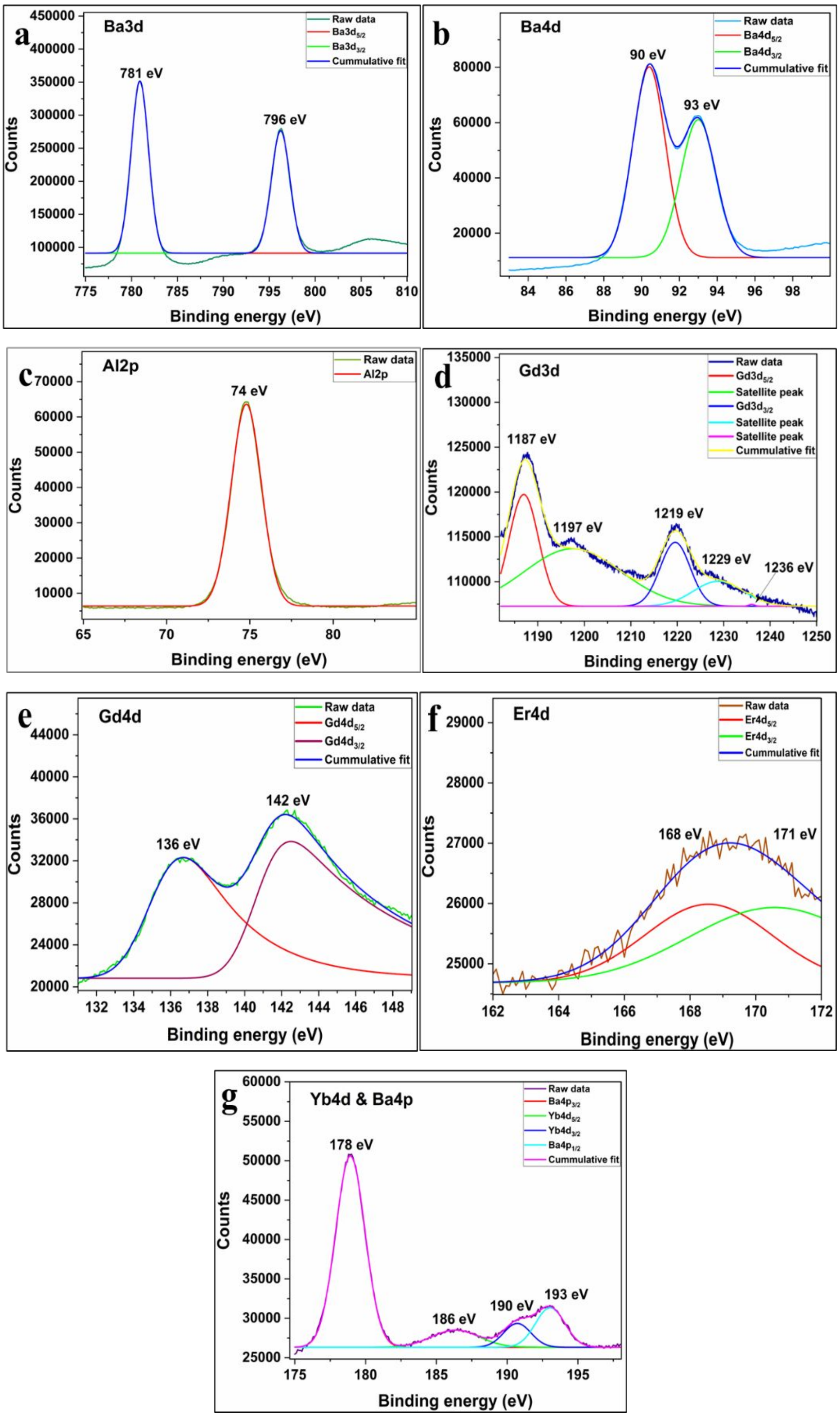

Figure S6: Full scan spectra of a) Ba3d, b) Ba4d, c) Al2p, d) Gd3d, e) Gd4d, f) Er4d and g) Yb4d & Ba4p.

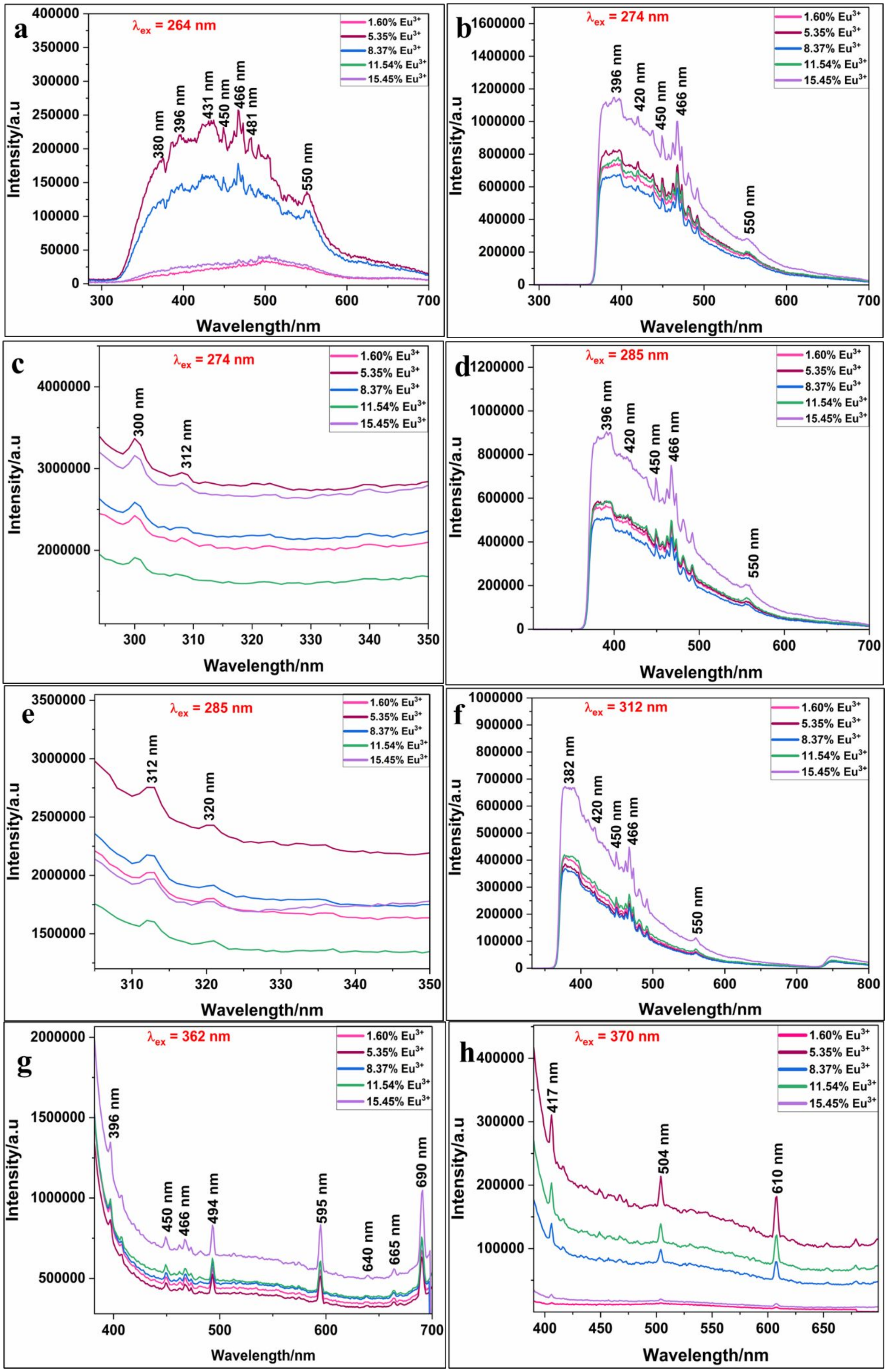
a
λex = 264 nm
1.60% Eu3+
5.35% Eu3+
8.37% Eu3+
11.54% Eu3+
15.45% Eu3+
380 nm
396 nm
431 nm
450 nm
466 nm
481 nm
550 nm
Intensity/a.u
Wavelength/nm
b
λex = 274 nm
396 nm
420 nm
450 nm
466 nm
550 nm
c
λex = 274 nm
300 nm
312 nm
d
λex = 285 nm
396 nm
420 nm
450 nm
466 nm
550 nm
e
λex = 285 nm
312 nm
320 nm
f
λex = 312 nm
382 nm
420 nm
450 nm
466 nm
550 nm
g
λex = 362 nm
396 nm
450 nm
466 nm
494 nm
595 nm
640 nm
665 nm
690 nm
h
λex = 370 nm
417 nm
504 nm
610 nm

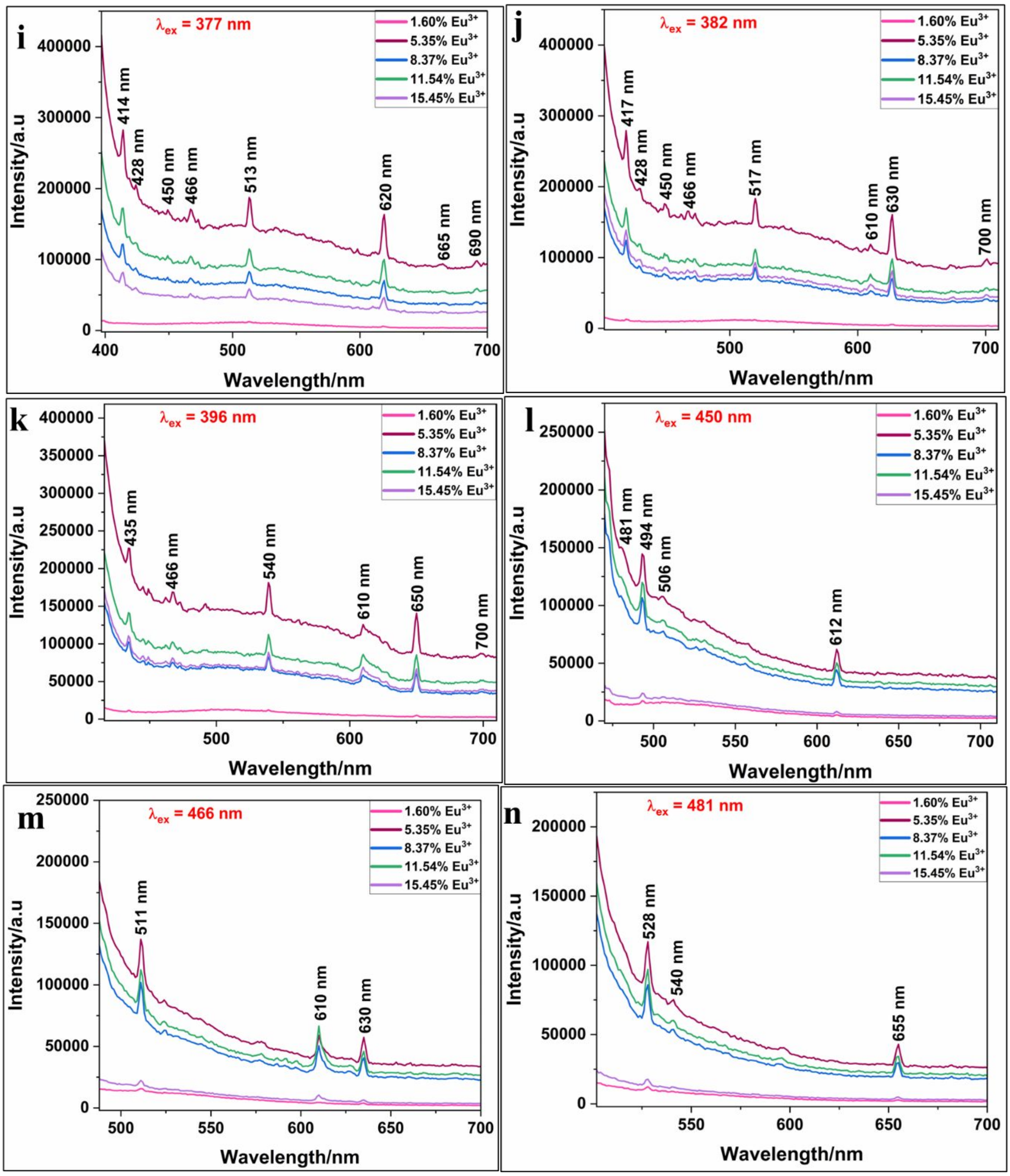

Figure S7: Photoluminescence spectra of various Eu^{3+} doped BAO phosphors recorded at a) 264 nm, b) & c) 274 nm with and without 370 nm optical filter, d) & e) 285 nm with and without 370 nm optical filter, f) 312 nm, g) 362 nm, h) 370 nm, i) 377 nm, j) 382 nm, k) 396 nm, l) 450 nm, m) 466 nm and n) 481 nm.

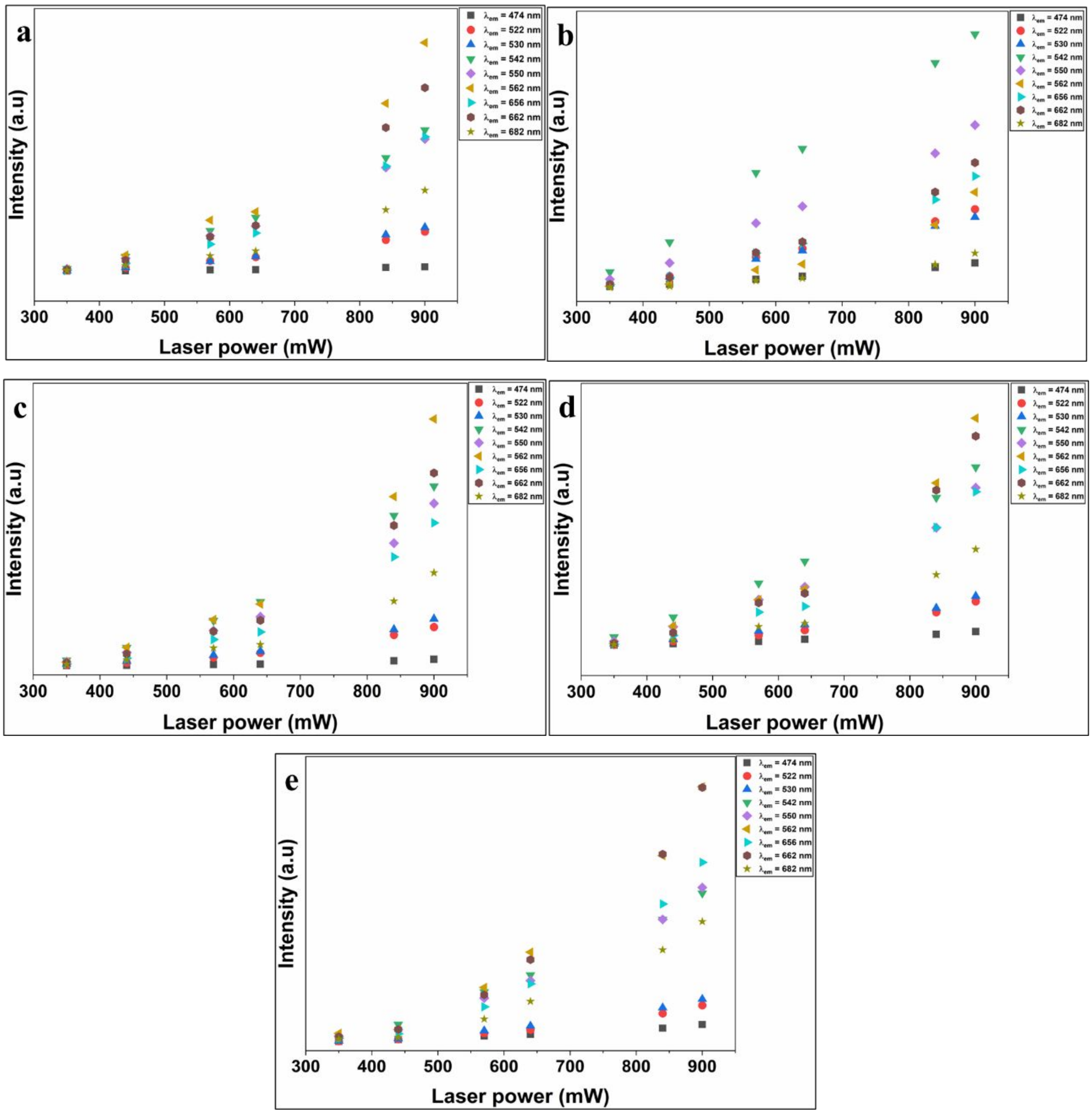

Figure S8: Variation of UC intensities of 474, 522, 530, 542, 550, 562, 656, 662 and 682 nm emission bands with increasing laser power for a) 1.60% Eu^{3+}, b) 5.35% Eu^{3+}, c) 8.37% Eu^{3+}, d) 11.54% Eu^{3+} and e) 15.45% Eu^{3+} doped phosphors.

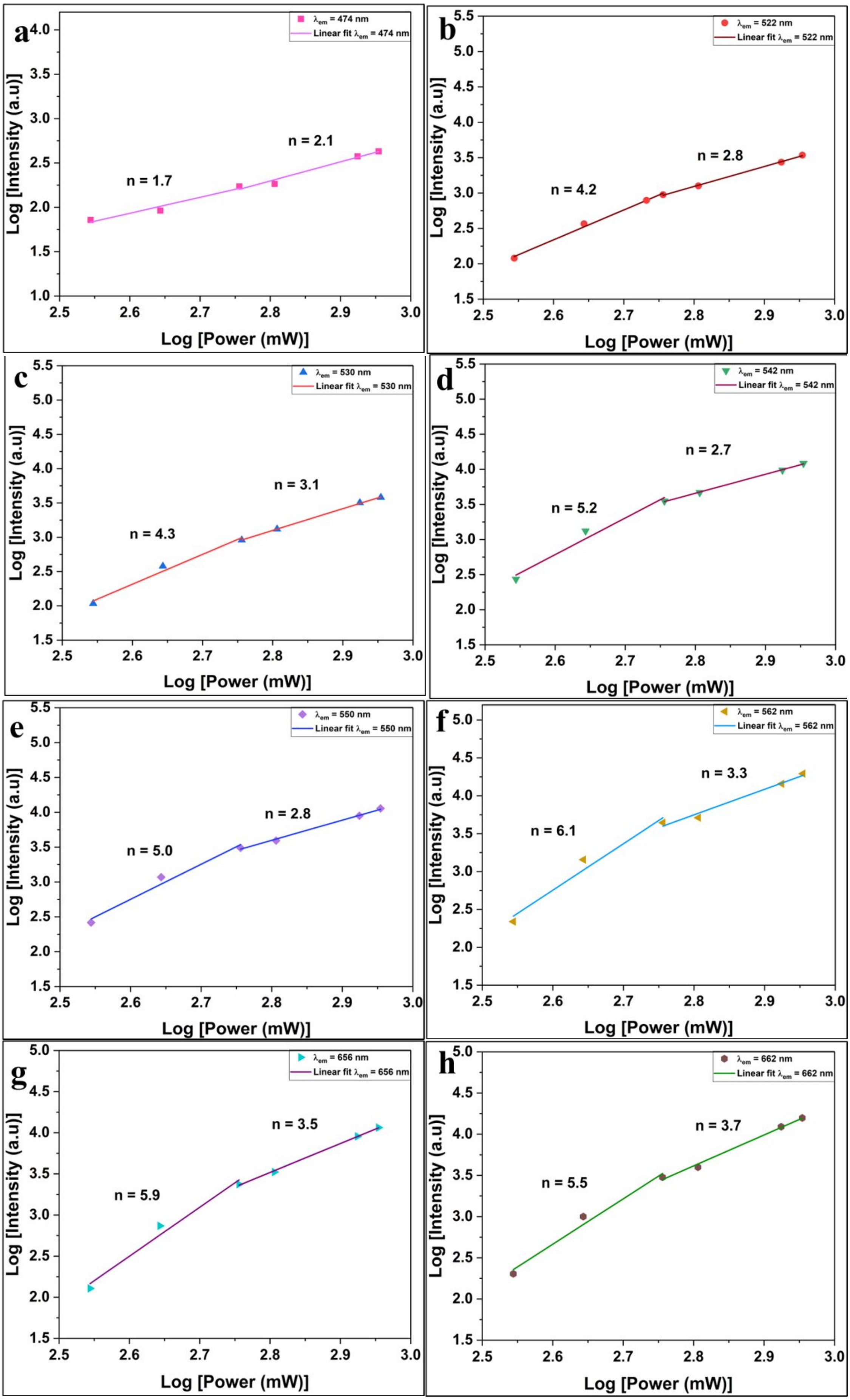

a
λem = 474 nm
Linear fit λem = 474 nm
n = 1.7
n = 2.1
b
λem = 522 nm
Linear fit λem = 522 nm
n = 4.2
n = 2.8
c
λem = 530 nm
Linear fit λem = 530 nm
n = 4.3
n = 3.1
d
λem = 542 nm
Linear fit λem = 542 nm
n = 5.2
n = 2.7
e
λem = 550 nm
Linear fit λem = 550 nm
n = 5.0
n = 2.8
f
λem = 562 nm
Linear fit λem = 562 nm
n = 6.1
n = 3.3
g
λem = 656 nm
Linear fit λem = 656 nm
n = 5.9
n = 3.5
h
λem = 662 nm
Linear fit λem = 662 nm
n = 5.5
n = 3.7
Log [Intensity (a.u)]
Log [Power (mW)]

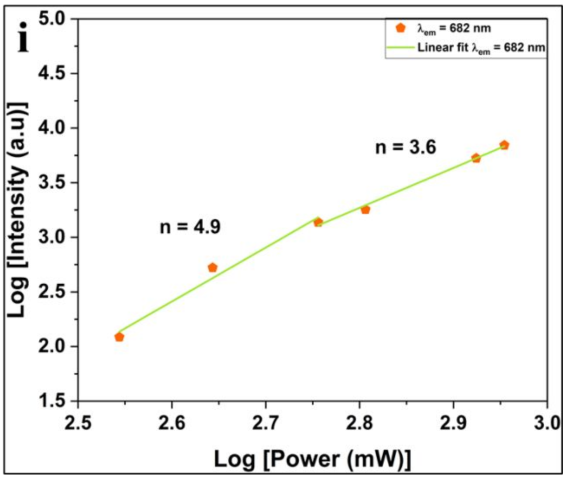

Figure S9: The logarithmic power-pump dependency on a) 474, b) 522, c) 530, d) 542, e) 550, f) 562, g) 656, h) 662 and i) 682 nm emission intensities for 1.60% Eu^{3+} doped BAO under 980 nm excitation.

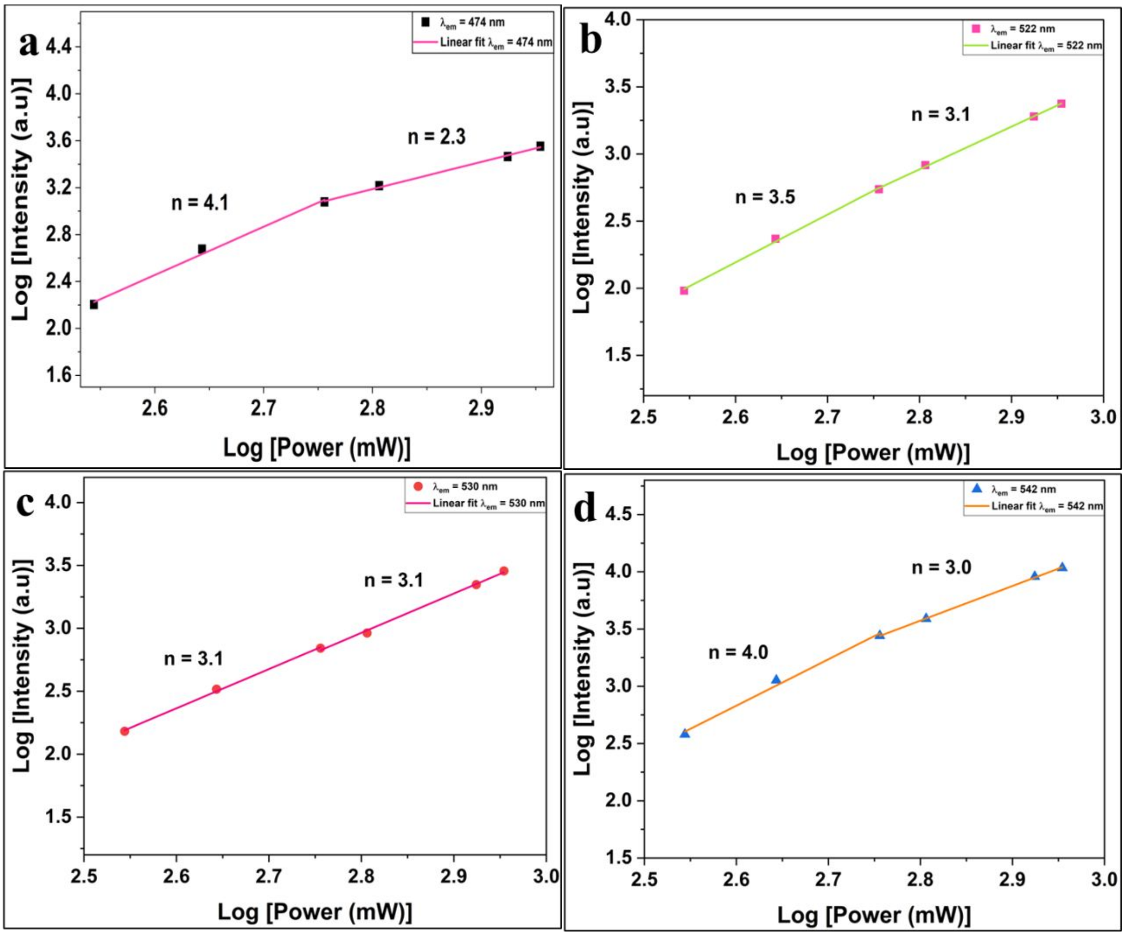

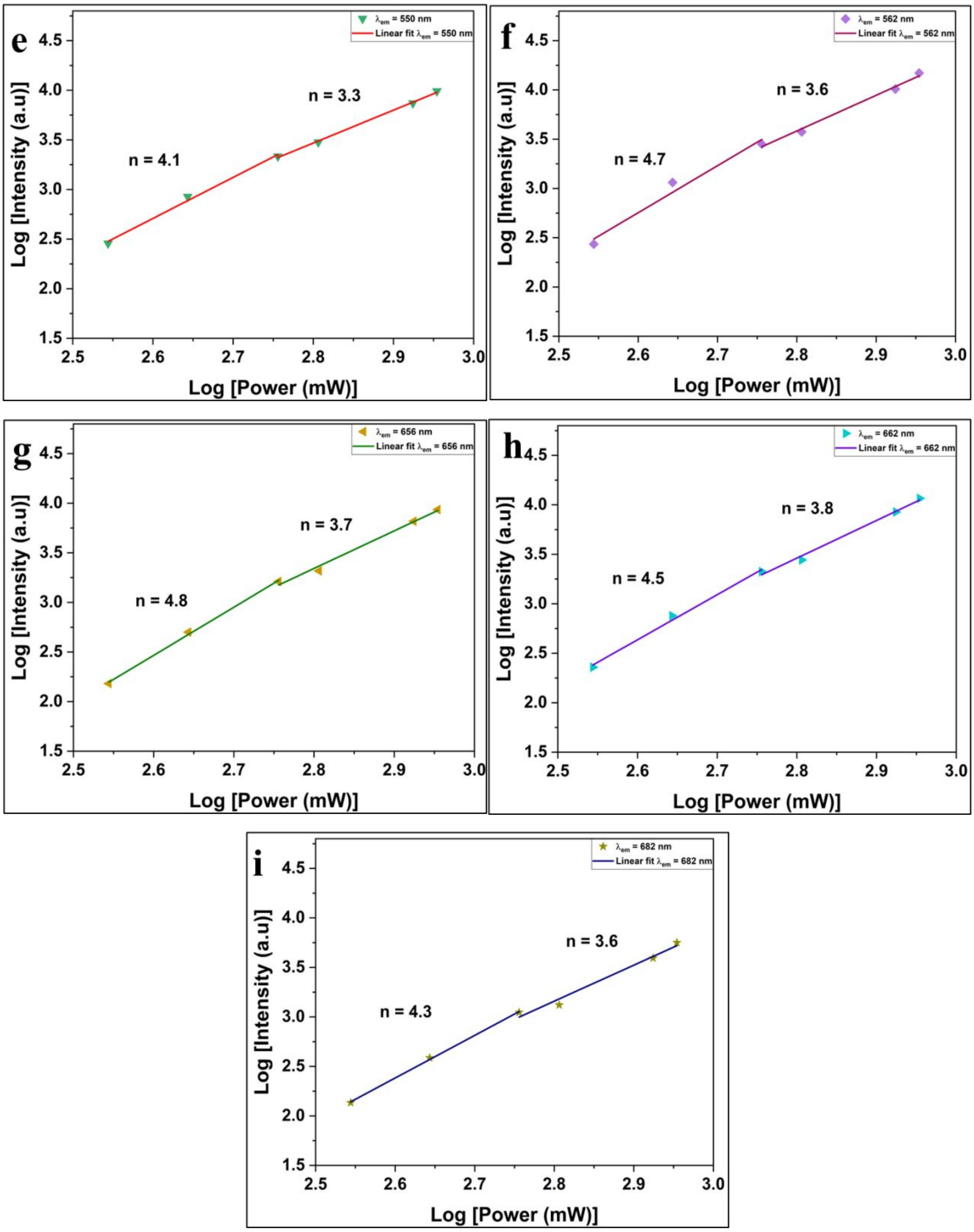

Figure S10: The logarithmic power-pump dependency on a) 474, b) 522, c) 530, d) 542, e) 550, f) 562, g) 656, h) 662 and i) 682 nm emission intensities for 5.35 % Eu^{3+} doped BAO under 980 nm excitation.

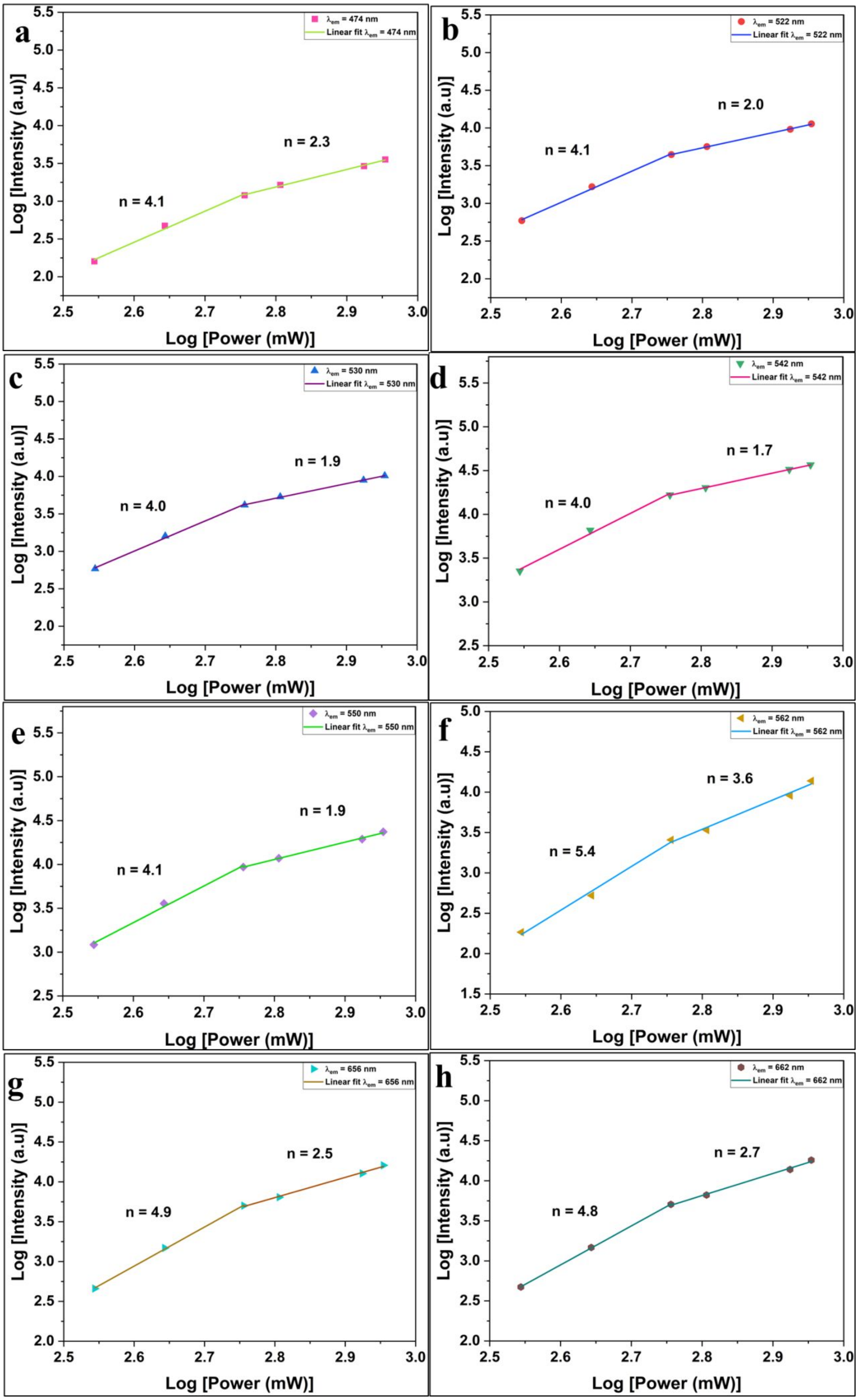

a
λem = 474 nm
Linear fit λem = 474 nm
n = 4.1
n = 2.3
b
λem = 522 nm
Linear fit λem = 522 nm
n = 4.1
n = 2.0
c
λem = 530 nm
Linear fit λem = 530 nm
n = 4.0
n = 1.9
d
λem = 542 nm
Linear fit λem = 542 nm
n = 4.0
n = 1.7
e
λem = 550 nm
Linear fit λem = 550 nm
n = 4.1
n = 1.9
f
λem = 562 nm
Linear fit λem = 562 nm
n = 5.4
n = 3.6
g
λem = 656 nm
Linear fit λem = 656 nm
n = 4.9
n = 2.5
h
λem = 662 nm
Linear fit λem = 662 nm
n = 4.8
n = 2.7
Log [Intensity (a.u)]
Log [Power (mW)]

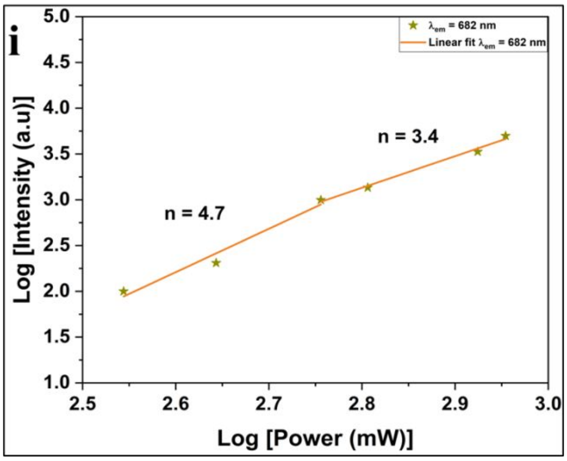

Figure S11: The logarithmic power-pump dependency on a) 474, b) 522, c) 530, d) 542, e) 550, f) 562, g) 656, h) 662 and i) 682 nm emission intensities for 8.37 % Eu^{3+} doped BAO under 980 nm excitation.

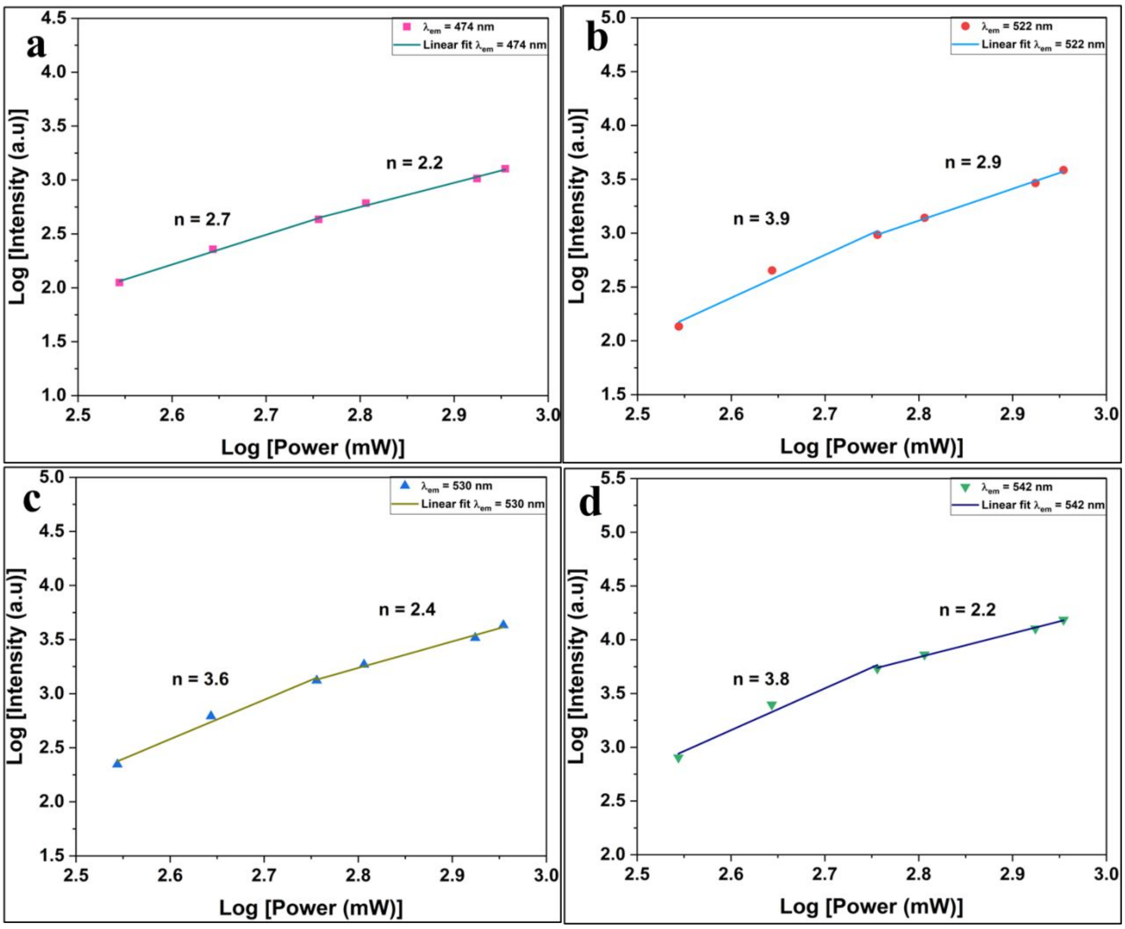

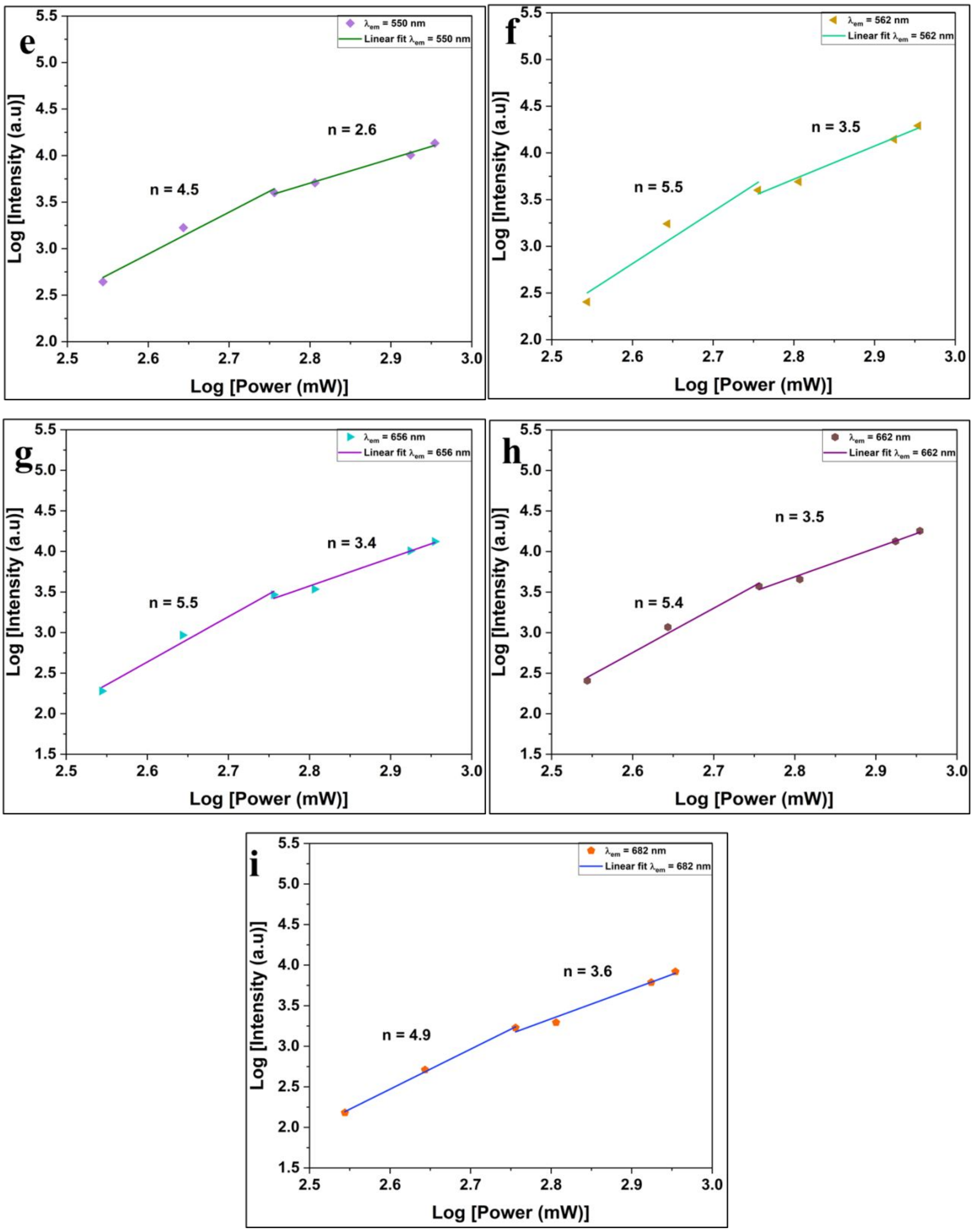

Figure S12: The logarithmic power-pump dependency on a) 474, b) 522, c) 530, d) 542, e) 550, f) 562, g) 656, h) 662 and i) 682 nm emission intensities for 11.54 % Eu^{3+} doped BAO under 980 nm excitation.

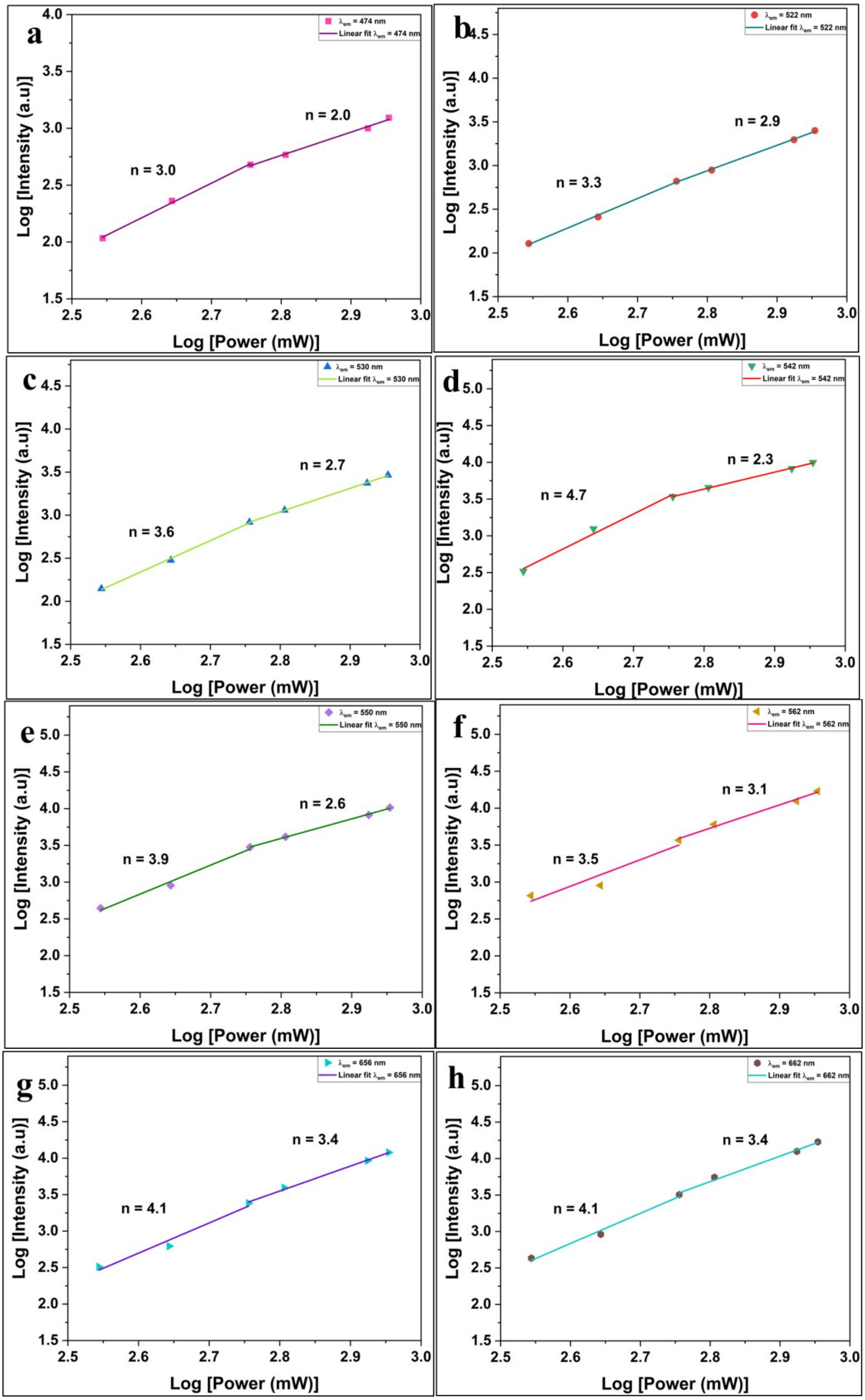
a
b
c
d
e
f
g
h
λem = 474 nm
Linear fit λem = 474 nm
λem = 522 nm
Linear fit λem = 522 nm
λem = 530 nm
Linear fit λem = 530 nm
λem = 542 nm
Linear fit λem = 542 nm
λem = 550 nm
Linear fit λem = 550 nm
λem = 562 nm
Linear fit λem = 562 nm
λem = 656 nm
Linear fit λem = 656 nm
λem = 662 nm
Linear fit λem = 662 nm
n = 3.0
n = 2.0
n = 3.3
n = 2.9
n = 3.6
n = 2.7
n = 4.7
n = 2.3
n = 3.9
n = 2.6
n = 3.5
n = 3.1
n = 4.1
n = 3.4
n = 4.1
n = 3.4
Log [Intensity (a.u)]
Log [Power (mW)]

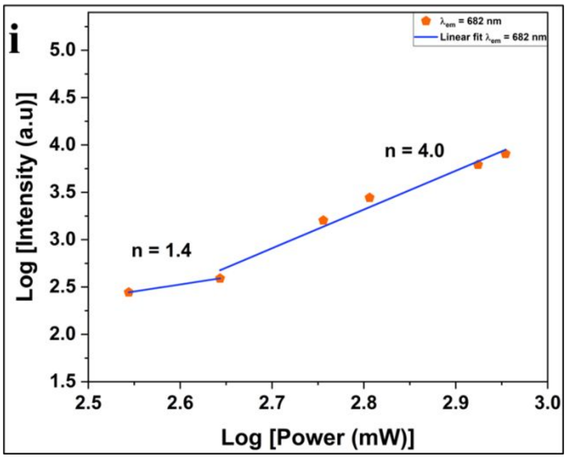

Figure S13: The logarithmic power-pump dependency on a) 474, b) 522, c) 530, d) 542, e) 550, f) 562, g) 656 h) 662, and i) 682 nm emission intensities for 15.45 % Eu^{3+} doped BAO under 980 nm excitation.

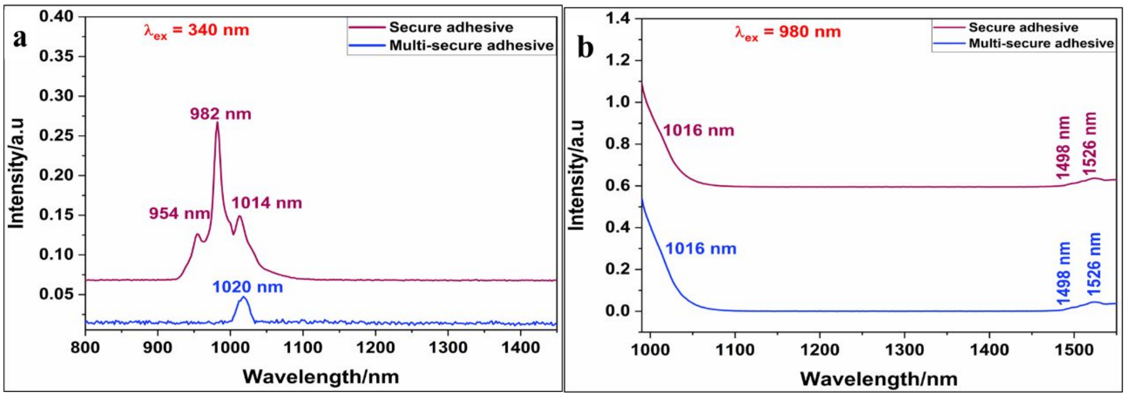

Figure S14: NIR emissions of adhesives recorded at a) 340 and b) 980 nm excitations.

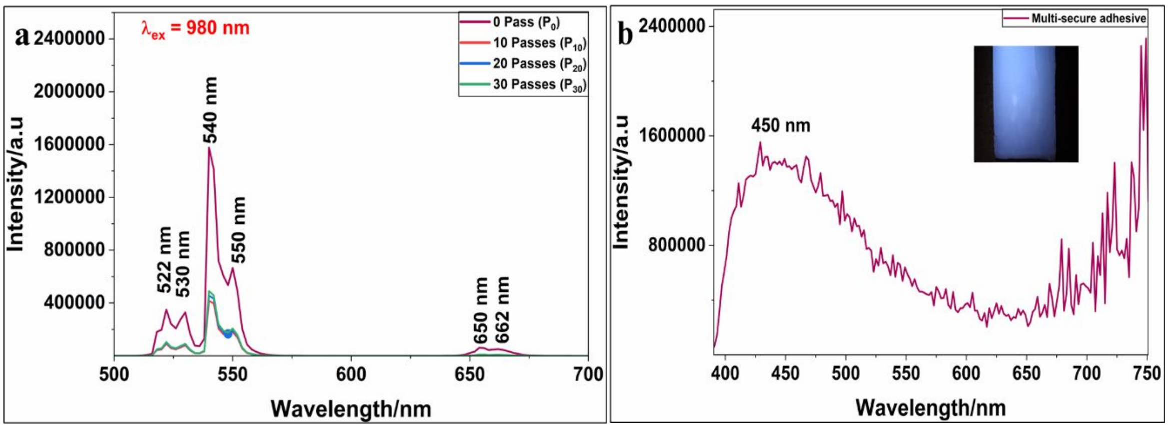

Figure S15: PL spectra of multi-secure adhesive sample exposed to different UV passage cycles. b) PL spectra for multi-secure adhesive at 365 nm excitation (inset image consisting multi-secure adhesive under 365 nm UV light).

Table S1: D-space value estimated using Bragg's equation for JUP-AS120.

SR.NO	2θ	Interplanar spacing
1	17.1	0.52
2	24.53	0.36
3	25.96	0.34
4	28.08	0.32
5	29.86	0.3
6	30.73	0.29
7	32.41	0.28
8	34.59	0.26
9	39.54	0.23
10	43.38	0.21
11	46.45	0.2
12	52.08	0.18
13	53.09	0.17
14	53.58	0.17
15	55.27	0.17
16	61.25	0.15
17	62.18	0.15
18	64.12	0.15
19	65.01	0.14
20	70.91	0.13
21	72.3	0.13
22	77.52	0.12

Table S2: D-space value estimated using Bragg's equation for BAO.

Peak number	2θ	d-space value
1	19.54	0.45
2	20.23	0.44
3	22.01	0.40
4	28.23	0.32
5	34.25	0.26
6	35.77	0.25
7	39.78	0.23
8	40.08	0.22
9	40.92	0.22
10	45.02	0.20
11	45.8	0.19
12	53.41	0.17
13	54.5	0.17
14	57.76	0.16
15	61.41	0.15
16	69.51	0.14

Table S3: Temperature dependent viscosity values for control, secure and multi-secure adhesives.

Temp (°C)	Viscosity (cP)		
	Control	Secure	Muti-Secure
90	9190	9904	10860
95	9040	9581	10590
100	8850	9074	9860
105	8420	8629	8810
110	7680	7410	7900
115	6830	7553	7670
